# Predicting anti-PD-1 immune checkpoint blockade response in melanoma patients with spatially aware machine learning models

**DOI:** 10.1038/s41698-025-01250-8

**Published:** 2026-01-12

**Authors:** Alyssa Pybus, Raphael Kirchgaessner, Jonathan Nguyen, Carlos Moran Segura, Paulo Cilas Morais Lyra, Trevor Rose, Jhanelle Gray, Jeremy Goecks, Joseph Markowitz

**Affiliations:** 1 Department of Machine Learning, H. Lee Moffitt Cancer Center & Research Institute, Tampa, FL USA; 2 Knight Cancer Institute, Oregon Health & Sciences University, Portland, OR USA; 3 Advanced Analytical and Digital Laboratory, H. Lee Moffitt Cancer Center & Research Institute, Tampa, FL USA; 4 Department of Radiology, H. Lee Moffitt Cancer Center & Research Institute, Tampa, FL USA; 5 Department of Thoracic Oncology, H. Lee Moffitt Cancer Center & Research Institute, Tampa, FL USA; 6 Department of Cutaneous Oncology, H. Lee Moffitt Cancer Center & Research Institute, Tampa, FL USA; 7 Department of Oncologic Sciences, University of South Florida, Morsani School of Medicine, Tampa, FL USA

**Keywords:** Biomarkers, Cancer, Computational biology and bioinformatics, Immunology, Oncology

## Abstract

There is an acute need to accurately identify patients with advanced melanoma who are most likely to respond to anti-PD1 immune checkpoint blockade (ICB) therapy. While anti-PD1 therapy can be highly effective in advanced melanoma patients, only 30-40% of patients respond well. In this study, we apply single-cell spatial proteomics together with statistical and machine learning (ML) methods to successfully predict advanced melanoma patient response to anti-PD1 ICB in a cohort of 12 patients with >8 million cells. While no single molecular feature is sufficient to predict ICB response in our cohort, ML models integrating multiple molecular features accurately predict response in 11 of 12 patients. A recurrent cellular neighborhood analysis revealed a tumor-infiltrating lymphocytes niche that was present in the tumors of most responders. This neighborhood, tumor microenvironment immune cell composition, and levels of nitric oxide synthases were all important features used by our ML models to make accurate predictions. Optimal predictive performance by our ML models—a ROC AUC of 0.76—was achieved when using all molecular features, including cellular spatial relationships, but limiting our analysis to only immune-rich tissue regions. This study demonstrates the feasibility of using machine learning models to accurately predict patient response to anti-PD1 ICB therapy using spatial proteomics datasets.

## Introduction

Patients with advanced melanoma (stage III and stage IV) show a 30–40% response rate to anti-PD-1 immune checkpoint blockade (ICB) drugs pembrolizumab and nivolumab^1–4^. Importantly, patients who respond to ICB often show a durable response to therapy that can last for years. For example, a recent follow-up of the KEYNOTE-006 trial reports that pembrolizumab has improved median melanoma-specific survival in advanced disease to over 4 years^3^. Unfortunately, there are no accurate biomarkers for predicting long-term response to immunotherapy in melanoma^5–9^. Well-studied biomarkers sometimes used by oncologists to assess the likelihood of melanoma patient response to ICB, such as tumor mutational burden^10^ (TMB) and PD-L1 expression^11^, have not been shown to be predictive. Given this landscape, there remains a tremendous opportunity to develop new approaches for predicting ICB response in advanced melanoma. Improving the predictive accuracy of ICB response would substantially improve the treatment of this patient cohort. Patients likely to respond to therapy could be given ICB as a first-line therapy, whereas patients unlikely to respond could pursue alternative therapy options earlier^12–15^.

New approaches to improve predictions of ICB response in advanced melanoma and other cancers have used a variety of omics approaches. Genomics, transcriptomics, proteomics, and even metabolomics and radiomics have been shown to have predictive power, and research in these areas is ongoing^16^. Recently, single-cell spatial proteomics has proven especially promising for understanding tumor microenvironments (TME) and linking TME features to clinical attributes such as patient response to therapy. Spatial proteomics has been used to make novel insights about the TME, shedding light on cellular heterogeneity, tumor progression, and tumor-immune dynamics across a wide range of cancers^17,18^. Several studies have found that spatial localization of T-cell infiltration and interactions between lymphocytes, macrophages, and PD-L1 within the tumor compartment can be used to predict melanoma patient response to ICBs^18–23^. Specifically, spatial co-localization of PD-L1+ cells and macrophages with CD8 + T-cells and tumor cells correlated with favorable outcomes to ICB therapy^20,21^. Machine learning (ML) has become an important tool for analyzing cancer spatial proteomics and transcriptomics datasets because these datasets are quite complex^24,25^. Recent applications of ML to TME spatial omics datasets include predicting transition to invasive breast cancer^26^, understanding and predicting tumor response to a novel immunotherapy^27^, and tailoring therapy based on predictions of tumor response to therapy^28^.

This study builds on spatial proteomics applications in cancer and uses statistical and ML methods to predict response to ICB response in advanced melanoma. We assayed pretreatment advanced melanoma biopsies using two 8-plex multiplex immunofluorescence (mIF) panels consisting of lymphoid or myeloid markers, nitric oxide synthases (iNOS, eNOS, nNOS), immune checkpoint markers (PD-L1, LAG-3), and malignant melanoma tumor cell marker SOX10. Nitric Oxide (NO) dependent processes are associated with either the death of melanoma cells or the progression of the disease^29^. Therefore, these panels were developed to investigate the dichotomy of NO in melanoma. Our data set includes more than 8 million cells from 12 advanced melanoma tumors from separate patients. We observe no strong univariate biomarkers that predict response, but ML models accurately predict ICB response for 11 of 12 patients in our cohort. A detailed analysis of >1700 1 mm^2^ tissue tiles reveals that the most accurate predictions are made using compositional and spatial information from immune-rich areas of the tumors. Our most accurate ML models have a receiver operating characteristic (ROC) area under the curve (AUC) of 0.76. The most important TME features used by the ML models to make accurate predictions were the immune and nitric oxide synthases features in immune-rich areas of the tumors. Our results demonstrate how targeted spatial proteomics analysis may play a role in precision medicine for advanced melanoma and future clinical trials.

## Results

### A study using single-cell spatial proteomics to assess ICB response in advanced melanoma

**Fig. 1:**
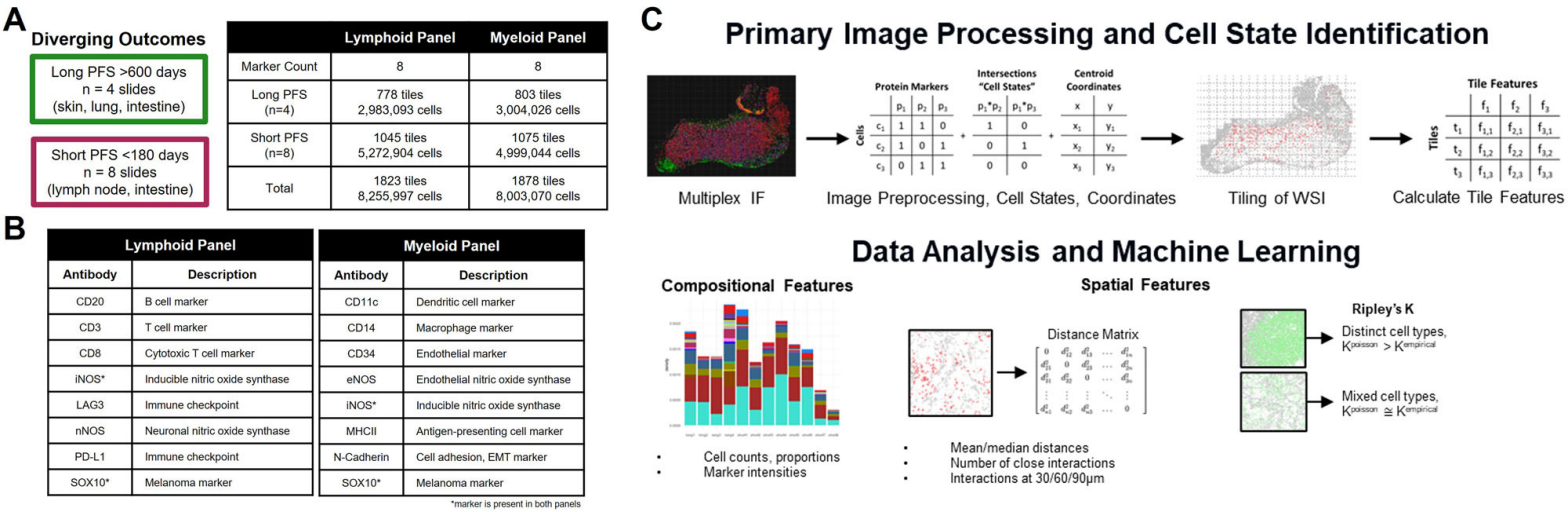
Experimental overview. Our study includes 12 advanced melanoma patients who underwent anti-PD1 immune checkpoint blockade (ICB) after tumor resection/biopsy. **A** Four patients experienced complete or partial response to ICB with >600 days of progression-free survival (“responders”) while eight were assessed as progressive disease with progression within 161 days of the start of treatment (“non-responders”). Tumor sections collected prior to treatment underwent multiplex immunofluorescence (mIF) with custom lymphoid and myeloid marker melanoma panels. Whole-slide images were sectioned into 1 mm by 1 mm tiles for analysis. **B** Protein markers included in each mIF panel. A variety of immune-related markers were probed as well as melanoma marker SOX10, cell adhesion marker N-Cadherin, and nitric oxide synthases iNOS, nNOS, and eNOS. **C** Whole-slide images underwent primary image processing, cell state assignment, and 1 mm by 1 mm image tiling (see Methods), followed by statistical and machine learning analyses using compositional features (cell state proportions and specific cell subpopulations in a tile) and spatial features (normalized counts of distances between cells within defined radii, Ripley’s K spatial statistics).

Our study cohort consists of twelve stage IV melanoma patients who underwent a surgical tumor resection or biopsy and then received anti-PD1 ICB therapy, either pembrolizumab or nivolumab (Table 1). Four patients experienced a progression-free survival (PFS) of >600 days with RECIST responses of complete or partial response. We term this group the ICB responder group. A non-responder group of eight patients showed tumor progression within at most 161 days (Fig. 1A). To assess the single-cell spatial structure of patient tumors, multiplex immunofluorescence (mIF) was performed on each patient resection or biopsy. Two custom mIF panels of eight markers each were used: a lymphoid panel (CD3, CD8, CD20, PD-L1, LAG-3, SOX10, iNOS, nNOS) and a myeloid panel (CD11c, CD14, CD34, MHCII, N-Cadherin, SOX10, iNOS, eNOS) (Fig. 1B). Together, these panels and markers provide single-cell spatial information on malignant cells, lymphoid and myeloid immune cells, and cells expressing nitric oxide synthase proteins.

**Table 1:** Cohort characteristics

Slide ID	Therapy	Response (RECIST)	Age at IO	Sex	Metastatic stage	PFS (Days)	Resection site
L1	Pembrolizumab	Partial response	61	M	M1c	911^a^	Intestine
L2	Pembrolizumab	Complete response	61	F	M1c	805^a^	Skin
L3	Pembrolizumab	Partial response	57	F	M1b	826^a^	Skin
L4	Nivolumab	Partial response	60	M	M1c	615^a^	Lung
S1	Pembrolizumab	Progressive disease	44	M	M1c	84	Lymph node
S2	Pembrolizumab	Progressive disease	41	F	M1c	148	Lymph node
S3	Pembrolizumab	Progressive disease	58	M	M1c	161	Intestine
S4	Pembrolizumab	Progressive disease	68	M	M1c	161	Intestine
S5	Pembrolizumab	Progressive disease	79	F	M1a	81	Lymph node
S6	Pembrolizumab	Progressive disease	66	M	M1c	155	Intestine
S7	Nivolumab	Progressive disease	64	F	M1c	79	Lung^b^
S8	Pembrolizumab	Progressive disease	69	M	M1c	84	Lymph node^b^

Demographic and treatment information for the study cohort of twelve stage IV melanoma patients at Moffitt Cancer Center. All patients received anti-PD1 immune checkpoint blockade (ICB) therapy (pembrolizumab or nivolumab) following tumor resection/biopsy. Four patients (L1-L4) experienced progression-free survival (PFS) of >600 days (all had ongoing PFS by the end of the study) while eight (S1-S8) showed progression within 161 days.

*RECIST* Response evaluation criteria in solid tumors.

^a^Indicates ongoing progression-free survival at time of study conclusion.

^b^Indicates core biopsies (S7/S8); all other samples are surgically resected tumors.

Images from the mIF panels underwent primary image processing, including cell segmentation and intensity thresholding to produce single-cell annotations for binary protein expression of each of the eight markers (Fig. 1C, Methods). Cell states were defined as the 20 most prevalent protein marker combinations among the entire cell population, plus expression of any single protein marker (see Methods). In total, 8,254,246 cells were identified across all patient samples, and 22 cell states were identified with the lymphoid mIF panel (8,000,383 cells and 20 cell states with the myeloid mIF panel). Cells lacking any of the eight protein markers are labeled as stromal cells because they are not defined as malignant or immune cells within the limitations of our panel. Lymphoid and myeloid mIF panels were analyzed separately.

We then tiled the images into smaller sections and applied statistical and ML approaches to understand and predict ICB therapy response differences between responders and non-responders in our cohort. To capture spatial information at the local level, we separated each whole slide image into several square tiles of 1 mm^2^ (Fig. 1C), totaling approximately 3 million cells across 699 tiles from the responder cohort and 5 million cells across 983 tiles from the non-responder cohort (Fig. S1). Each tile was characterized using both compositional and spatial features. Compositional features include (a) proportions of cell states within each tile, such as the proportion of CD3 + CD8+ cells in a tile, and (b) proportions of marker positivity within a cell state, such as the proportion of CD8+ cells all CD3+ cells in a tile. Tile spatial features quantified include average distances between cell states, the relative number of cell state pairs within specific distances (20, 30, 60, 90 µm), and measures of cell state mixing using Ripley’s K spatial statistics (variance-stabilized to Ripley’s L). Finally, we used statistical and ML methods to analyze tile compositional and spatial features to quantify differences between responders and non-responders (Fig. 1C). The differences identified by these quantitative approaches provide insight into mechanisms of response for anti-PD-1 ICB therapy in advanced melanoma and suggest approaches for predicting response to ICB therapy.

### Characterizing compositional and spatial cellular features among responders and non-responders

**Fig. 2:**
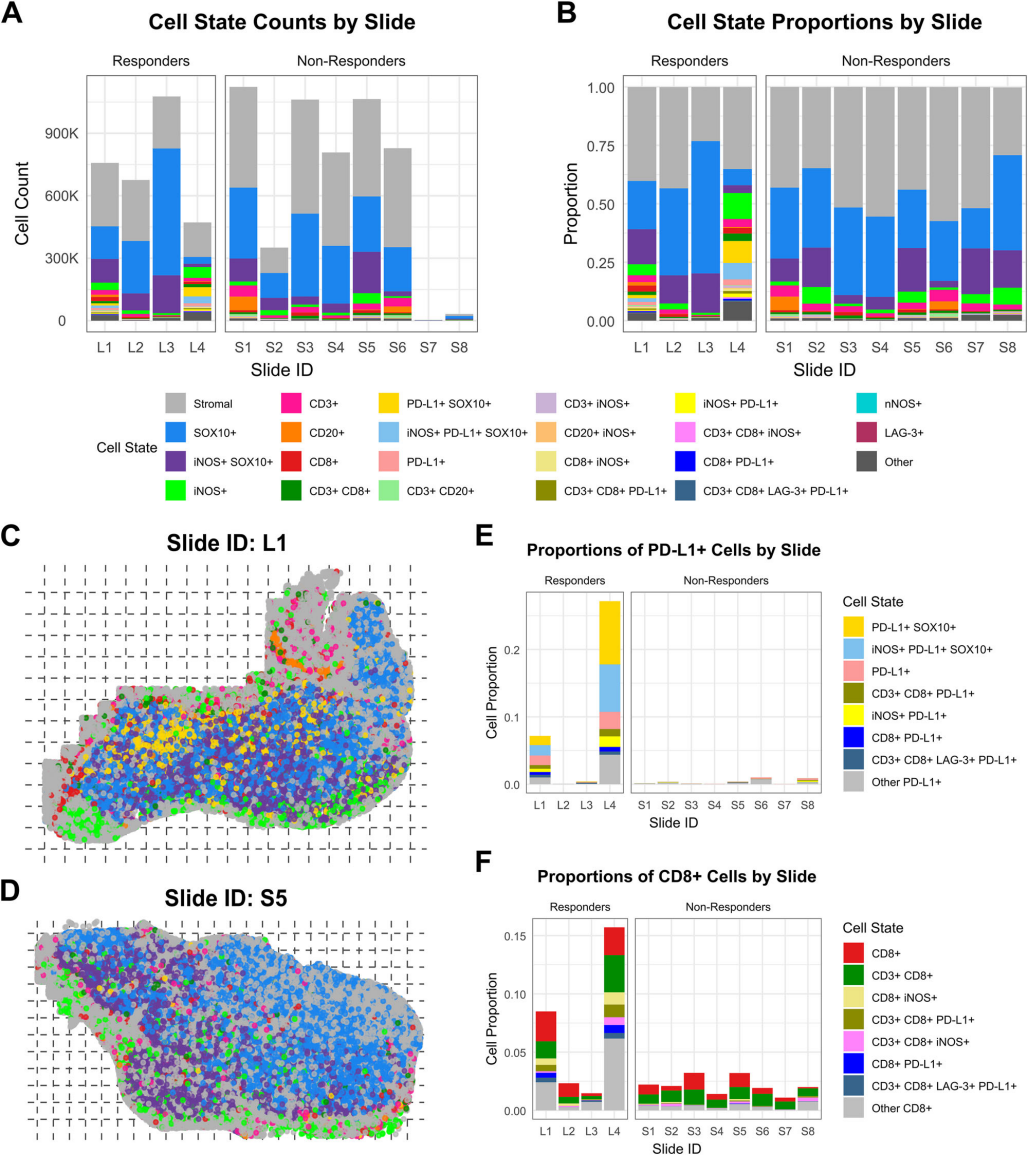
Cell state composition by slide, lymphoid mIF panel. Stacked bar charts displaying cell counts (**A**) and proportions (**B**) of each of 22 cell states (plus an “Other” category) by slide. Bars representing ICB responders are displayed on the left (L1-L4) of each chart, and bars for non-responders are on the right (S1-S8). Cells lacking any of the eight protein markers are labeled Stromal. Whole-slide visualizations of the spatial distribution of the top 8 cell states (Stromal, SOX10+, iNOS+ SOX10+, iNOS+, CD3+, CD20+, CD8+, CD3 + CD8+) and any PD-L1+ cells (yellow) for one responder sample (**C**, slide L1) and one non-responder sample (**D**, S5). Cells are plotted by their computed centroids. Dashed lines indicate tile x and y limits. Tiles are 1 mm by 1 mm in size. Stacked bar charts displaying cell state proportions for all cell states containing PD-L1 (**E**) or CD8 (**F**) by slide. All cells which contain PD-L1 or CD8 but are not captured within the 22 defined cell states are represented in gray for “Other PD-L1 + ” or “Other CD8 + ”. Elevated proportions of PD-L1+ and CD8+ cells are found in the immunoreactive responder samples (L1/L4) compared to immune-cold responders (L2/L3) and non-responders (S1–S8).

To evaluate patient-to-patient variation in cell state composition, we first quantified cell state counts and proportions at the whole-slide level (Fig. 2A–D for the lymphoid panel, Fig. S2 for the myeloid panel). Total cell counts and proportions varied widely from slide to slide, and the two biopsies (S7, S8) had very few cells. Among responders, we noted two “immunoreactive” samples (L1, L4) with high PD-L1+ and CD8+ cell proportions and two “immune-cold” ICB responders (L2, L3). All non-responder samples were immune cold as well (Fig. 2E, F). No single cell state proportion was statistically significantly different between responders and non-responders, including PD-L1+ cells (Fig. 2E, F for lymphoid panel, Figs. S2 and S3 for myeloid panel, and additional lymphoid details). Visual spatial distributions of top cell states for each mIF panel did not reveal any clear patterns (Fig. S4 for the lymphoid panel and Fig. S5 for the myeloid panel). Tertiary lymphoid structures (TLS) were not observed in any of the 12 patient samples.

**Fig. 3:**
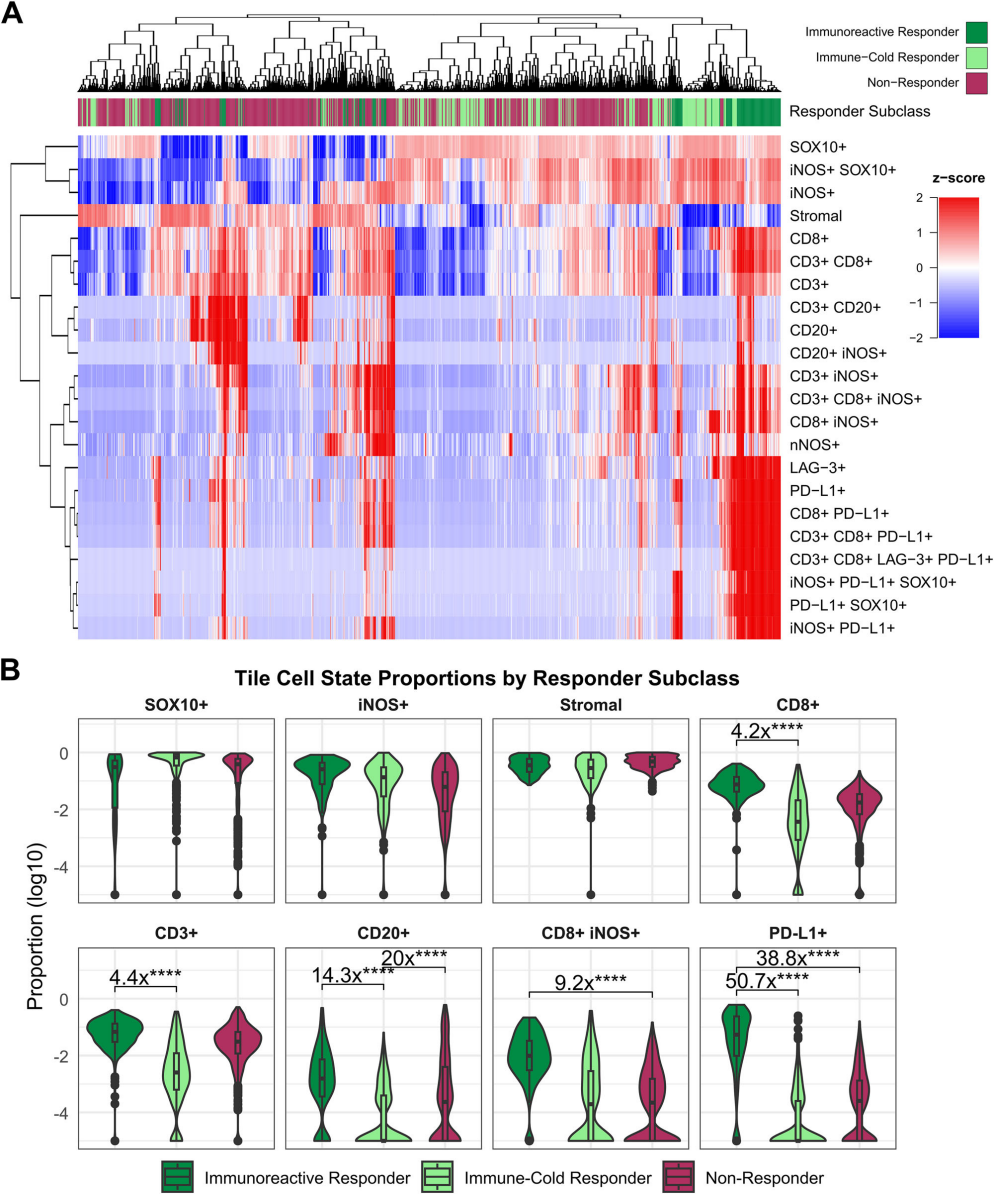
Cell state compositional tile features distinguish immunoreactive responders, immune-cold responders, and non-responders to ICB therapy. Lymphoid panel mIF whole slide images from each patient are separated into 1 mm by 1 mm tiles, and proportions of each of 22 defined cell states are calculated within each tile. **A** A heatmap displaying the relative proportions of each cell state (rows) within all 1682 tiles (columns). Proportions are z-scored across rows to represent the relative expression of each protein (red to blue for high to low expression). The color bar above the heatmap denotes the ICB response subclass of each sample: Immunoreactive Responders (L1/L4), Immune-Cold Responders (L2/L3), and Non-Responders (S1–S8). Rows and columns are clustered by Euclidean distance. Tiles from ICB responders generally cluster separately from those of non-responders, though major differences exist between immunoreactive and immune-cold responder tiles. **B** The tile proportions of SOX10+, iNOS+, Stromal, CD8+, CD3+, CD20+, CD8+ iNOS+, and PD-L1+ cell states (left to right, top to bottom) are displayed according to ICB response subclass: Immunoreactive Responders (L1/L4), Immune-Cold Responders (L2/L3), and Non-Responders (S1–S8). Significant differences (FDR-corrected Wilcoxon rank-sum *p*-values) with a fold-change of 4x or greater between response classes are displayed (****p < 0.0001). As most markers show differing distributions between immunoreactive and immune-cold responder tiles, no single marker proportion shows the ability to distinguish between all responders (immunoreactive and immune-cold) and non-responders.

Next, we assessed compositional and spatial tile features to capture trends at the local tissue level. Hierarchical clustering of cell state proportions within 1 mm^2^ tiles across all samples showed separation of most ICB responder tiles from most non-responder tiles (Fig. 3A, immunoreactive and immune-cold responder tiles cluster to the right), indicating potential for pre-treatment ICB response classification from the combined information across our 22 defined cell states of the lymphoid mIF panel. In concordance with the slide-level analysis, we found several cell state proportions that distinguish the two immunoreactive responders’ tiles from the tiles of non-responders and immune-cold responders (Fig. 3A). Key distinguishing features include cell state proportions containing cytotoxic T-cell marker CD8 and immune checkpoint marker PD-L1 (Fig. 3B). However, these cell state proportions alone fail to distinguish all ICB responders from non-responders because some responders are immunoreactive while some are immune-cold, similar to non-responders. Hierarchical clustering of cell state proportions from the myeloid mIF panel showed poorer separation by tile response class (Fig. S6A) and revealed several elevated cell state proportions in immunoreactive responder tiles versus immune-cold responders and non-responders, including CD14+, NCad+, and eNOS+ cells (Fig. S6B). Conversely, MHCII+ cell proportions were elevated in non-responder tiles.

We next conducted a correlation analysis between individual protein markers in both responder and non-responder tiles to determine co-occurring or mutually exclusive cell type pairs (Fig. S7). Within the lymphoid panel data (Fig. S7A, B), responders showed increased correlation between tile proportions of CD8+ cells and both PD-L1+ (*R* = 0.5 vs *R* = 0.27) and LAG-3+ cells (*R* = 0.53 vs *R* = 0.31) compared to non-responders (Fig. S7B, left two plots), suggesting co-localization of CD8+ cytotoxic T cells with immune checkpoint markers PD-L1 and LAG-3 in the pre-treatment TME contributes to successful ICB response. We also note a higher positive correlation of B cell marker CD20 with T cell marker CD3 cell proportions in non-responder tiles (*R* = 0.61 vs *R* = 0.44, Fig. S7B, rightmost plot), indicating a potential immunosuppressive role for B cells in the non-responder population in the absence of TLSs. Correlations from the myeloid panel data (Fig. S7C, D) showed higher correlation of cell adhesion marker NCad with monocyte/macrophage marker CD14 (*R* = 0.72 vs *R* = 0.06), nitric oxide synthase eNOS (*R* = 0.21 vs *R* = −0.04), and dendritic cell marker CD11c (*R* = 0.22 vs *R* = −0.08) in responder tiles compared to non-responder tiles (Fig. S7D).

To identify the significant features distinguishing ICB responders and non-responders, we conducted statistical testing using the Wilcoxon rank-sum test, which treats tiles as independent observations, as well as a mixed effects model that accounts for slide-to-slide variation among tiles. While caution is needed in interpreting the results of a Wilcoxon rank-sum test due to violation of the independent samples assumption, this approach allows for descriptive characterization of differences between ICB responders and non-responders at the local tissue level. We then complement these results with a mixed effects model, which accounts for slide-to-slide variation to assess potential population-level differences across our limited sample set.

**Fig. 4:**
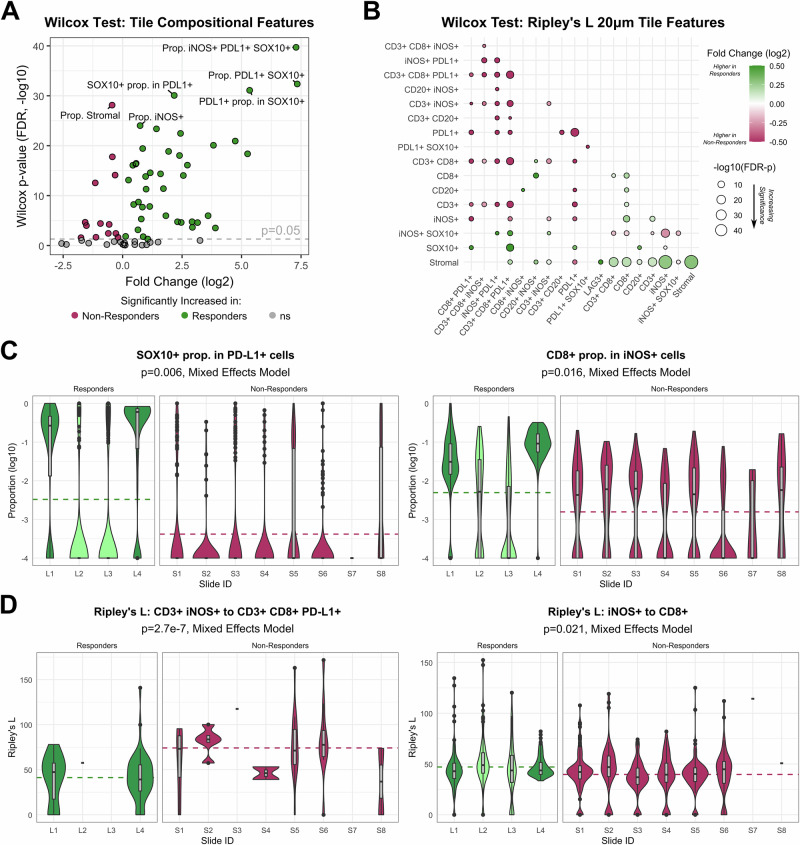
Univariate analysis of ICB responders vs non-responders reveals differentially expressed compositional and spatial tile features. Wilcoxon rank-sum test (**A**, **B**) and mixed effects modeling (**C**, **D**) were performed to identify compositional and spatial tile features from lymphoid panel mIF data in pre-treatment tumor samples capable of distinguishing ICB responders and non-responders. **A** Wilcoxon rank sum test was applied to 72 compositional tile features, including cell state proportions within the total tile population and proportions of each marker within subpopulations of each other marker. *P*-values were corrected for false discovery rate (FDR) using the Benjamini–Hochberg procedure. The volcano plot shows increasing significance on the vertical axis (-log10 of the FDR-adjusted *p*-values) and effect size on the horizontal axis (log2 of the mean fold-change). Positive fold-change values represent features increased within the ICB responder group. **B** Wilcoxon rank-sum test was applied to 168 Ripley’s L spatial tile features calculated at 20 µm distances between cells, and *p*-values were FDR-adjusted as above. The bubble plot shows bubbles of increasing size for increasing significance (-log10 of the FDR-adjusted *p*-values), colored by effect size (log2 of the mean fold-change, with green and maroon indicating increased values in responder or non-responder tiles, respectively) for cell state pairs indicated on the horizontal and vertical axes. Only significant differences are represented with a bubble (if a bubble is missing in the lower diagonal, *p* > 0.05). **C** To account for slide-to-slide variation within tiles, we also created mixed effects models using slide ID as a random effect to be controlled. Out of 72 compositional features, 8 resulted in *p* < 0.05, though none remained significant after FDR-adjustment. The tile distributions of the top two significant features are displayed by slide and response group on the horizontal axis and log10(proportion) values on the vertical axis. Dotted lines denote the average value of the feature across all tiles of each response class. Mixed effects model *p*-values displayed in the subtitles are unadjusted. **D** Same as **C** but for Ripley’s L spatial features calculated at 20 µm distances. Out of 62 features, 5 resulted in *p* < 0.05 with just Ripley’s *L* values between CD3+ iNOS+ and CD3 + CD8 + PD-L1+ cells remaining significant after FDR-adjustment (*p* = 2.7e-7, FDR-*p* = 1.7e-5). Only Ripley’s *L* features with non-missing values in at least 10 of the 12 slides were included in the analysis.

Wilcoxon rank-sum analysis of ICB responder versus non-responder tiles revealed 54 significantly different compositional features (Fig. 4A). Cell proportions higher in responder tiles as compared to non-responder tiles include iNOS+ PD-L1 + SOX10+ cells (*p* = 2.0e-40, 157x fold change), PD-L1 + SOX10+ cells (*p* = 4.3e-33, 162x fold change), iNOS+ cells (*p* = 9.4e-25, 1.7x fold change), and CD8+ iNOS+ cells (*p* = 3.4e-23, 5.4x fold change) (all Wilcox *p*-values are adjusted for false discovery rate using the Benjamini Hochberg procedure). Cell proportions higher in non-responder tiles include stromal cells (*p* = 7.5e-29, 1.4x fold change), CD3+ cells (1.7e-18, 1.2x fold change), and CD3 + CD20+ cells (*p* = 1.0e-4, 2.9x fold change). We also found 71 significantly different Ripley’s L 20 µm spatial features (Fig. 4B). In responder tiles, there is increased clustering of stromal cells between other stromal cells (*p* = 1.8e-46, 1.2x fold change), iNOS+ cells (*p* = 2.5e-45, 1.2x fold change), CD8+ cells (*p* = 7.8e-18, 1.1x fold change), and CD3+ cells (*p* = 1.3e-15, 1.1x fold change) compared to non-responder tiles. On the other hand, clustering at 20 µm was increased in non-responder tiles between iNOS+ cells and iNOS+ SOX10+ cells (*p* = 9.2e-15, 1.2x fold change), PD-L1+ cells with each other (*p* = 4.0e-14, 2.0x fold change), and CD3 + CD8 + PD-L1+ cells with each other (*p* = 6.6e-11, 2.4x fold change) and CD3 + CD8+ cells (2.8e-10, 1.6x fold change).

Compositional features most different between responder and non-responder tiles from the myeloid panel include the proportion of MHCII+ cells in CD14+ cells (*p* = 1.2e-55, 19.7x fold change), the proportion of MHCII+ cells (*p* = 3.6e-54, 8.0x fold change), and the proportion of MHCII+ cells in CD11c+ cells (*p* = 5.7e-44, 4.5x fold change), all increased in non-responder tiles (Fig. S8A). Spatial features of top significance in the myeloid panel also show increased Ripley’s L clustering of stromal cells with various other cell types (eNOS+, CD34+ eNOS+, CD14+, iNOS+, CD34+ cells) in responder tiles, and we also observed significantly increased clustering of NCad+ cells with NCad+ SOX10+ cells (*p* = 3.1e-14, 1.6x fold change) in non-responder tiles, among other features (Fig. S8B).

To account for the effect of slide-to-slide variation, we also calculated mixed effects models using slide ID as a random effect and each tile feature as a fixed effect, one model per feature. The two top compositional features distinguishing responders (*n* = 4) from non-responders (*n* = 8) in the mixed effects model include the proportion of SOX10+ cells within the PD-L1+ subpopulation (*p* = 0.006) as well as the proportion of CD8+ cells within the iNOS+ subpopulation (*p* = 0.016), though neither comparison remained significant after correction for false-discovery rate (FDR) among the 72 examined compositional features (Fig. 4C). Among Ripley’s L spatial features, we found significantly increased clustering within 20 µm between CD3+ iNOS+ cells and CD3 + CD8 + PD-L1+ cells in non-responders compared to responders (*p* = 2.7e-7, FDR-*p* = 1.7e-5), with no other features remaining significant after FDR-adjustment (Fig. 4D). Although not significant after adjustment, we observed trends of increased clustering between iNOS+ and CD8+ cells in responders compared to non-responders. Similar mixed effects modeling of features from the myeloid panel did not reveal and significant differences between response groups, though we observed trends of increased proportions of CD14 + CD34+ cells and CD34+ proportion in SOX10+ cells within responder tiles (Fig. S8C) and trends of increased stromal to eNOS+ cell clustering in responder tiles and increased CD34+ to CD14 + SOX10+ cell clustering in non-responder tiles (Fig. S8D).

### Recurrent cellular neighborhood analysis reveals tumor-infiltrating lymphocytes niche in ICB responders

**Fig. 5:**
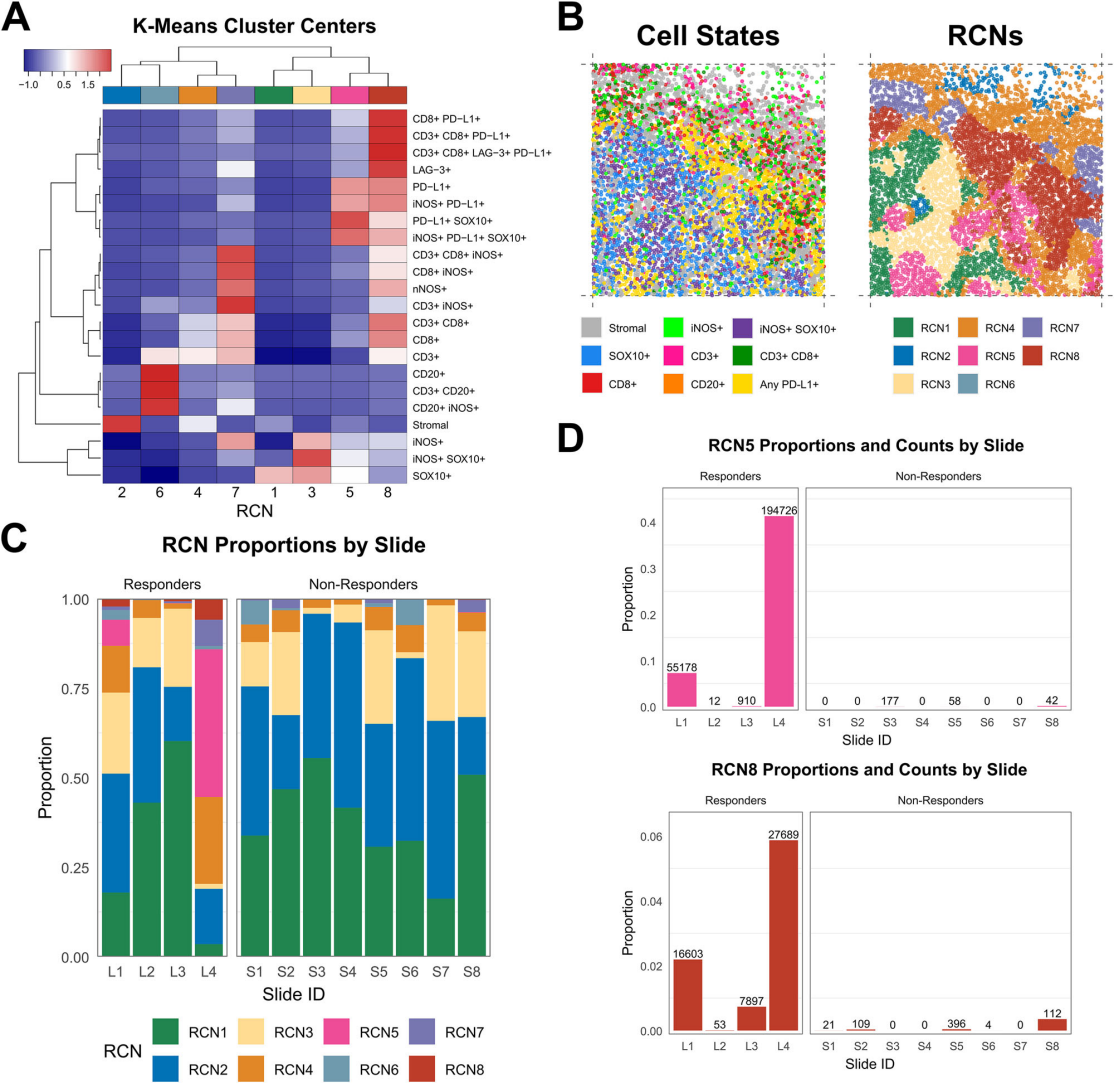
Recurrent cellular neighborhoods analysis. Recurrent cellular neighborhoods (RCNs) were determined via k-means clustering of all lymphoid mIF panel cellular neighborhoods across 12 slides. **A** K-means clustering resulted in eight distinct cluster centers, which define representative cell state proportion compositions for each RCN. Each column represents an RCN, while rows represent cellular neighborhood cell state proportions. Red to blue indicates relatively high to low expression of the corresponding cell state proportion within each RCN. Rows and columns are clustered using Euclidean distance. RCNs are labeled according to size, with RCN1 representing the largest cluster (3,172,458 neighborhoods) down to RCN8, the smallest (52,884 neighborhoods). **B** A single tile from slide L1 shown with cell states (left) and computed RCN labels (right). **C** Stacked bar chart showing the proportion of cellular neighborhoods within each slide corresponding to each RCN label. Bars representing ICB responders are displayed on the left (L1-L4), and bars for non-responders are on the right (S1-S8). **D** Bar charts showing the proportion of RCN5- and RCN8-labeled cellular neighborhoods within each slide with labels for total count of neighborhoods displayed above.

To quantify local tissue spatial architectures, we conducted a recurrent cellular neighborhood (RCN) analysis across all tumor samples. Neighborhoods were defined as the collection of all cells with centroids within 60 µm of a central index cell, in concordance with similar studies examining tumor immune microenvironment cell-cell interactions^27,30,31^. We defined neighborhoods for each of the ~8 M total cells in our dataset, calculated proportions of each cell state within each neighborhood, and then conducted k-means clustering to identify recurrent patterns (see Methods). Our analysis of the lymphoid mIF panel yielded eight distinct RCN (Fig. 5A, B) that were present in varying degrees across all twelve patients (Fig. 5C, Fig. S9). In concordance with the PD-L1+ cell state proportions in Fig. 2B, there was strong enrichment of the PD-L1-enriched cluster RCN5 in the immunoreactive responders (L1/L4) but very limited representation of this RCN among immune-cold responders (L2/L3) and non-responders (Fig. 5D). Interestingly, RCN8 proportions among all neighborhoods within each patient corresponded well with ICB response status: three ICB responders showed the highest RCN8 proportions (Fig. 5D). RCN8 proportions are significantly increased in responders compared to non-responders at the tile level (Wilcoxon rank-sum, *p* = 1.1e-13). Average RCN8 proportions by slide are higher in responders as well (2.2% vs 0.053%), though this comparison is not statistically significant given our small sample size. RCN8 is enriched for the cytotoxic T-cell marker CD8, the immune checkpoint markers LAG-3 and PD-L1, the malignant melanoma marker SOX10, and the enzyme iNOS, representing potential regions of tumor-infiltrating lymphocytes (TILs). RCN analysis of the myeloid mIF panel data created clusters of distinct combinations of cell types (Fig. S10A) but did not show differential enrichment of RCNs by patient response class (Fig. S10B, C).

### Machine learning models accurately predict patient ICB response

To build accurate predictive models of patient ICB response that use all available features, we developed and evaluated 13 ML model architectures. Three sets of features were used to create three distinct kinds of ML models: 1) “Compositional” models that use only compositional features—proportions and subpopulation proportions—for predictions; 2) “Combined” models that use compositional features and spatial features—RCN proportions and Ripley’s L between cell states at 20 µm—for predictions; and 3) “Immune-High” models that, due to our findings on the importance of “TILs RCN” in the previous section, uses compositional and spatial features from a subset of tiles with high proportions (≥0.02) of CD8+ cells for making predictions.

Figure S11 provides performance information from our assessment of different model architectures, spatial feature subset addition, and immune-high tile filtering, which informed our final model design.

We evaluated our models using Leave-One-Group-Out Cross–Validation (LOGO-CV), leaving one patient (group) out for each fold of cross–validation over 100 random iterations, resulting in 1200 training-validation splits per model type. Performance metrics were computed for each LOGO-CV iteration by aggregating predictions from every left-out patient set (see Methods). SHAP^32^ was used to estimate the importance of features in making predictions for each ML model. Amongst the 13 ML models evaluated on the combined feature set, the Gaussian Naïve Bayes (GaussianNB) model had optimal accuracy (0.69), ROC-AUC (0.65), and F1 (0.78) scores. However, the GaussianNB model displayed very low specificity (0.36), meaning that it often incorrectly predicted a lack of tumor response to therapy. We therefore selected the Light Gradient-Boosting Machine (LightGBM) model^33^ for use in all predictive models because LightGBM showed similar accuracy (0.67), ROC AUC (0.63), and F1 (0.74), as well as much higher specificity (0.50).

**Fig. 6:**
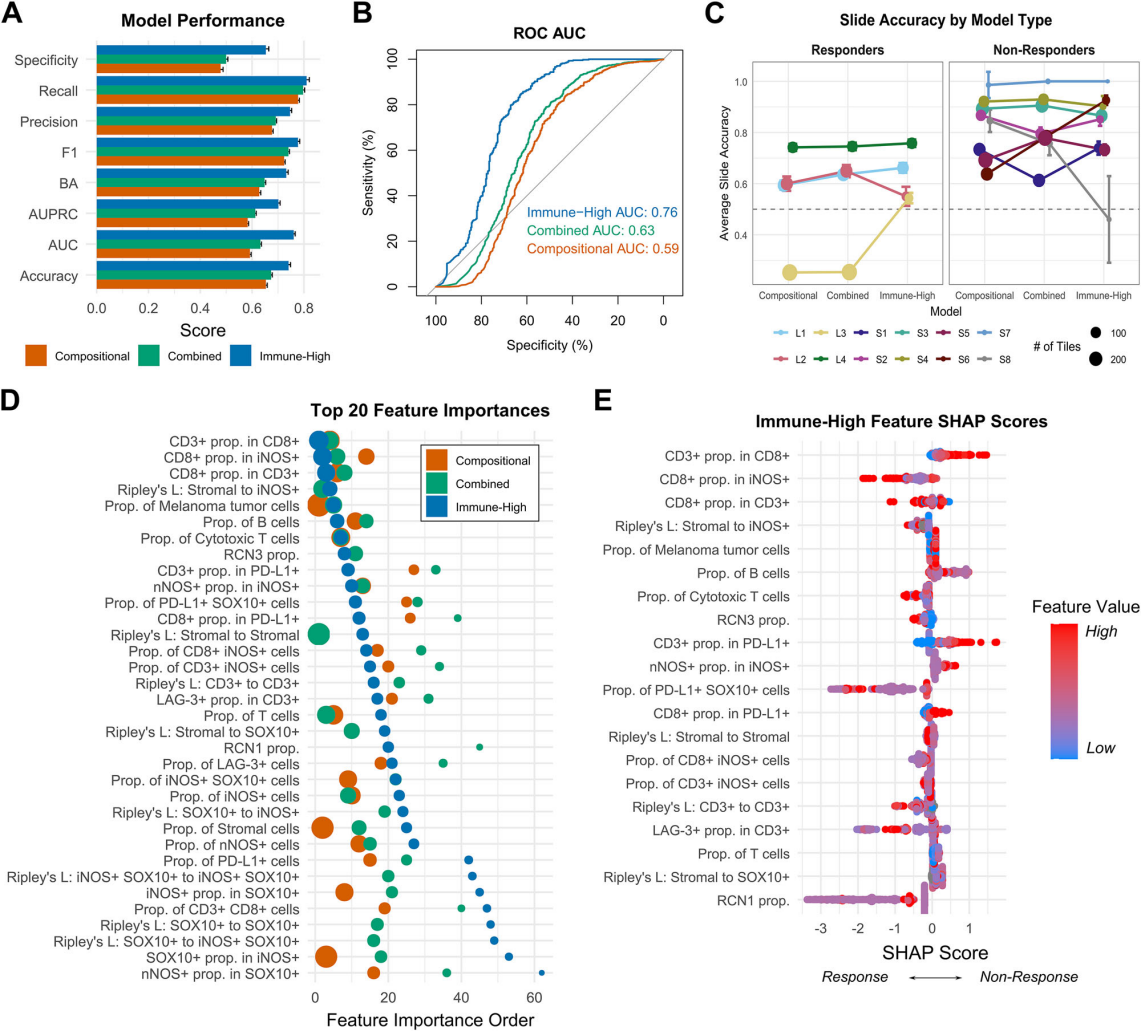
Models trained on immune-high tiles show improved classification performance and increased importance of immune-related features. Machine learning model performance metrics calculated from 100 iterations of 12-fold leave-one-out cross validation (LOOCV, leaving one of 12 patients out each time) for the (1) compositional feature only all-tile models (“Compositional”), (2) the combined compositional and spatial feature all-tile models (“Combined”), and (3) the combined compositional and spatial feature 2% CD8-filtered tiles model (“Immune-High”) with data from the lymphoid mIF panel. **A** Across all performance metrics, the Immune-High model type performs best followed by the Combined then Compositional models. Bars and error bars represent the average and standard deviation of each metric across 100 iterations of 12x LOOCV. **B** The receiver operating characteristic (ROC) curve shows model tradeoffs between sensitivity (true positive rate) and specificity (true negative rate). Area under the curve (AUC) represents the model’s ability to distinguish between classes, with higher values corresponding to better performance. The Immune-High model shows the highest AUC (AUC = 0.76), followed by the Combined (AUC = 0.63) and the Compositional models (AUC = 0.59). **C** Tile accuracy by slide for each model type. Points represent average accuracy and error bars represent standard deviation across all 100 iterations of each leave-one-out model. Point size represents the relative number of tiles used for the model test set, with smaller tile sets for the immune-high model (includes only tiles with ≥2% CD8+ cells). The dashed gray line indicates an accuracy of 50%. If slide prediction of patient ICB response is determined by the majority class prediction of its constituent tiles, the immune-high model is the only model which correctly predicts all tumor resection samples (L1–L4, S1–S6), though it usually incorrectly classifies S8, a core biopsy from a non-responder. **D** The order of average feature importance values for each model type (Compositional, Combined, and Immune-High). Data points are colored by model type and sized by their relative average feature importance value. A feature importance order of 1 indicates the feature had the highest feature importance value within that model type. All features within the top 20 average feature importance for any model type are shown. **E** Shapley Additive exPlanation (SHAP) scores from a representative Immune-High model iteration of 12x LOOCV show the relative effect (SHAP score, *x*-axis) of each feature (*y*-axis) alongside its relative feature value (red to blue indicates high to low feature values) for each predicted tile (data points). Negative to positive SHAP scores indicate that the associated feature value pushed the model towards predicting ICB response or non-response, respectively, while SHAP scores close to zero indicate little to no effect on prediction. Prop. of Melanoma tumor cells = SOX10+, Prop. of B cells = CD20+, Prop. of Cytotoxic T cells = CD8+, Prop. of T cells = CD3+.

The Combined model outperformed the Compositional model with consistent and moderately better performance in all metrics (Fig. 6A). These results demonstrate the value of incorporating spatial features with compositional features as Combined model performance was 0.01–0.04 higher than the Compositional model, with the receiver operating characteristic curve (ROC AUC) increasing from 0.59 to 0.63 (Fig. 6B). The addition of spatial features resulted in significantly increased patient-specific tile prediction accuracy for 2 of 4 responders and 5 of 8 non-responders (*p* < 0.05, Wilcoxon rank-sum; Fig. 6C). Notable compositional features appearing in the top 10 average feature importances for both Compositional and Combined model types included the proportions of stromal cells, malignant cells, nitric oxide synthase iNOS+ cells, T cells, and cytotoxic T cells (Fig. 6D, E). Important spatial features included Ripley’s L statistics for the self-clustering of stromal cells; the clustering of stromal cells with iNOS + , SOX10+ (melanoma), and CD3+ cells; the self-clustering of iNOS+ melanoma cells; and the self-clustering of melanoma cells (Fig. 6D, E). These findings demonstrate the importance of both compositional and spatial information among immune cells, iNOS+ cells, and stromal cells in the TME for making accurate predictions of patient response.

We hypothesized that the T cell microenvironment may be especially informative of ICB response because anti-PD-1 ICB targets the PD-1 protein on the surface of T cells and because prior work found that T-cell subsets are predictive of response to immunotherapies^34^. To test this hypothesis, we trained an “Immune-High” model with only tiles above a certain proportion of the cytotoxic T cell marker CD8. A recent study has proposed a density threshold of 223 CD8+ cells/mm^2^ within the TME for predicting improved response to ICB^35^. We therefore experimented with CD8+ cell tile percentage cutoffs between 0 and 5% (densities between an average of 177 and 539 cells/mm^2^) and found optimal model performance for a 2% threshold (Fig. S11D). We then compared the performance of our Immune-High model with our Combined model that used all tiles. The Immune-High model performed substantially better than the Combined model **(**Fig. 6A, B**)**, including a 0.13 ROC AUC increase to 0.76 compared to 0.63 for the Combined model as well as a 0.15 improvement in specificity to 0.65 as compared to 0.50 for the Combined model.

In principle an alternative Immune-High predictive model could be built using features from tissue labeled as recurrent cellular neighborhood 8 (RCN8). However, our dataset s not large enough to evaluate this alternative model. Recall that RCN8 is associated with patient response to therapy and rich in CD8+ (Fig. 5D), so RCN8 provides another way to identify Immune-High tissue that may be useful for training a predictive model. However, three non-responder tumors in our cohort do not contain any RCN8 neighborhoods, and filtering our tissue tile set based on RCN8 instead of CD8 would result in removing almost half of non-responder samples. Arbitrarily selecting tissue tiles from samples without any RCN8 neighborhoods and setting RCN8 levels to zero is possible, but doing this would lead to the creation of a model that does not use only immune-rich regions and would violate the model's primary goal. The CD8 filter that we used enables building and evaluating an Immune-High model on our entire cohort.

We made patient-level response predictions by using the majority prediction for a patient’s tiles. For the Immune-High model, only tiles meeting the CD8+ cells proportion threshold were used. Patient-level prediction accuracy was significantly improved in the Immune-High vs Combined model for 3 of 4 responders and 3 of 8 non-responders. Accuracy substantially increased for samples S1 and L3 and decreased for only one core biopsy sample, S8 (fold change >1.2x, *p* < 0.001). Accuracy was minimally changed for 7 patients (fold change <1.1x), including the other core biopsy S7 (Fig. 6C). Importantly, “Immune-High” was the only model capable of attaining ≥50% tile accuracy for all ICB responders as well as all but one non-responder. The incorrectly predicted non-responder was a core biopsy with only six immune-high tiles (S8). The “Immune-High” model revealed increased importance of cells with co-expression of T cell markers CD3 and CD8, increased importance of the immune checkpoint marker PD-L1, and decreased importance of stromal cell information relative to the Combined and Compositional models (Fig. 6D, E). Together, these results suggest that immune-active regions contain especially important compositional and spatial information for determining a patient’s likelihood of response to ICB.

To ensure that excluded cell states beyond the top 20 threshold were unlikely to significantly influence model performance, we trained new ML models using compositional features (cell state proportions) of all cell states for each panel (199 in the lymphoid panel, 152 in the myeloid panel). Performance metrics were calculated from the average of 10 randomized model training iterations. Model accuracy when using “all cell states” compositional models was equal to (lymphoid panel, both 65%) or worse than (myeloid panel, 49% vs 52%) our reported parsimonious models. While it is possible a more accurate comprehensive model exists using additional compositional or spatial features, this is unlikely because (1) variance of the excluded features is low compared to included features, and low variance features often do not substantially impact model results (Figs. S12 and 2) we anticipate that spatial features using rare populations will not have a large impact on model accuracy as rare populations did not have a large impact on the compositional model (Fig. S13). Additionally, inclusion of all compositional features may not be desirable due to model overfitting, which may produce less accurate results.

Lastly, we trained and evaluated ICB response prediction models using the myeloid mIF panel data (Fig. S14). Myeloid panel models performed worse than lymphoid panel models, especially when using compositional features alone. Similar to our findings with the lymphoid models, the addition of spatial information improved all measured performance metrics (Fig. S14A, B) and most tile accuracy rates by slide (Fig. S14C). Top features included stromal cell clustering among themselves and with tumor cells (SOX10+) as well as proportions of CD34+ cells, macrophage (CD14+), iNOS+ cells, and antigen-presenting cells (MHCII+) (Fig. S14D, E).

## Discussion

There is an acute clinical need to better predict patient response to ICB in advanced melanoma and understand the biological mechanisms associated with ICB response. In this study of pre-anti-PD1 therapy stage IV melanoma patients, we generated a spatial single-cell targeted proteomics dataset using a novel multiplex immunofluorescence (mIF) panel. Statistical analyses of this dataset revealed novel TME features associated with anti-PD1 response, and our ML models correctly predicted patient response in 11 of 12 patients. Our study yielded three key findings. First, ML models that combined multiple TME features to predict patient response outperformed predictions made using all single TME features, including one current standard of care biomarker, PD-L1 positivity^36–38^. Second, our analysis demonstrates the importance of spatial analysis in classifying ICB response. A RCN resembling a tumor-infiltrating lymphocytes (TILs) niche was elevated in three of four ICB responder patients, and several spatial features showed significant differences in ICB responders and non-responders. Third, our ML models were much more accurate in predicting ICB response when trained only on immune-rich regions. Tile-level classification accuracy jumped from 67% to 74%, and ROC AUC rose from 0.63 to 0.76. ML models trained on immune-rich regions accurately predicted response in all four patients that responded to ICB therapy and in seven of eight patients that did not respond to therapy. Therefore, this model is potentially useful for predicting both responses and failures and additional cohorts should be analyzed prior to application in the biomarker directed clinical trial setting.

While current FDA-approved biomarkers for ICB response in melanoma do not consider spatial tumor organization (PD-L1 expression, TMB), our work and other recent studies show the potential of incorporating spatially derived biomarkers for improving prediction of response. Previous studies have identified spatial relationships between T cell and macrophage subtypes with various immune checkpoint markers and tumor cell markers^18,20–23^. In our study, the TIL-like RCN elevated in three of four ICB responder patients was enriched for cytotoxic T cell marker CD8, immune checkpoint markers LAG-3 and PD-L1, malignant melanoma tumor cell marker SOX10, and inducible nitric oxide synthase iNOS. Similarly, correlation of tile cell state proportions suggested increased co-localization of cytotoxic T cells with immune checkpoint markers LAG-3 and PD-L1 in responders compared to non-responders. These results are concordant with several studies highlighting the potential of using tumor-infiltrating lymphocytes as a predictive biomarker of ICB response in melanoma^39,40^. We also found significantly increased clustering of SOX10+ cells with PD-L1 + T cells in ICB responders, mirroring a recent study showing close interactions between tumor cells and PD-L1 + T cells are associated with favorable ICB response^21^. Lastly, our ML models performed substantially better when including spatial features (0.59–0.63 AUC in lymphoid, 0.52–0.60 AUC in myeloid), with the greatest performance improvement observed for spatial features computed at a close distance (20 µm).

There are no current standard-of-care biomarkers for ICB response in melanoma. PD-L1 and TMB are insufficient because there are many patients who respond to ICB despite low PD-L1 and/or TMB levels^10,15,41,42^. The shortcomings of single-biomarker predictions of ICB response likely stem from high tumoral heterogeneity typical of advanced melanoma^36–38^. In our cohort, responders included both those with “immunoreactive” TMEs characterized by high PD-L1 and CD8 expression but also those with “immune-cold” TMEs that more closely resembled non-responders. Our analysis shows that the integration of multiple features leads to a more accurate prediction of ICB response prediction. Unsupervised hierarchical clustering of lymphoid mIF panel TME features correctly grouped most tiles from ICB responders to a single cluster, while any single feature proved insufficient for grouping. In addition, our ICB response ML classifier integrated and weighted TME features to obtain 92% patient accuracy, with optimal performance achieved when training the classifier only on immune-rich tissue regions. This improvement suggests similar models or biomarkers may benefit from limiting analysis to immune-rich regions of the TME, potentially removing noise from less informative regions.

Nitric oxide and nitric oxide synthase have been implicated in both pro- and anti-tumor effects based on concentration and the cell type of expression^29,43–47^. Within our cohort, iNOS expression in cytotoxic T cells was associated with ICB response as indicated by significantly increased cell type proportions by our univariate analysis as well as high feature importance within our ICB response ML model. We also found increased levels of iNOS+ tumor cells were associated with response. Spatially, an iNOS+ malignant tumor recurrent cellular neighborhood (RCN3) emerged within the top 10 most important features, with higher proportions associated with anti-PD-1 response. Together, these findings support evidence of iNOS as a potential pre-treatment biomarker of anti-PD-1 response in melanoma, particularly when expressed within T cells. It is noted that there is extensive literature of iNOS being a poor prognostic factor for melanoma patients due to its effects on the TME as reviewed elsewhere^29^, though initial studies were done in the era prior to checkpoint blockade^48^. Further research is needed to fully elucidate its dichotomous role in tumor progression and/or immune activation both before and during immunotherapy.

While our current study is limited to advanced melanoma patients receiving anti-PD-1 therapy, our use of spatial proteomics together with ML is generalizable and can be applied to studies of other ICB monotherapies like anti-CTLA-4 and anti-LAG-3 as well as combination ICB therapies in future work. We chose to first study anti-PD-1 response as it forms the backbone of modern melanoma treatment and has clinical relevance in decision making^1,49–53^. Specifically, a key clinical question when treating advanced melanoma is whether single-agent therapy is sufficient for an individual patient or whether combination therapy, which carries a greater risk of adverse events, is needed. To study combination ICB therapy it is unknown at this time whether new mIF panels will be needed. Ultimately, a risk stratification tool that assists in selecting between single-agent ICB therapy and combination therapy for treating advanced melanoma would be very valuable.

A notable limitation of our study is the small cohort size of ICB responder patients (*n* = 4). This limitation is magnified by the high level of variability of several key features within the responder cohort (the dichotomy between immunoreactive and immune-cold responders, particularly in PD-L1 and CD8 expression), variations in tumor collection site (skin, intestine, lung, and lymph node), and the use of resections plus core-needle biopsies. However, the wealth of data and our tiling approach to focus on local tissue microenvironments—14 unique protein markers, several hundred spatial features, more than 8 million cells, and ~1700 tiles—allow for meaningful analysis despite our small patient cohort. Future work should expand on our exploratory study to validate these findings with larger patient populations. This work is also limited by the relatively small number of protein markers within a single mIF panel, preventing detailed immune cell subtyping and quantification of spatial proximity between both lymphoid and myeloid cell types.

Another limitation of this study is its inability to assess the stromal cell population. Recall that cells not expressing any of the measured proteins were labeled as stromal cells, and in several of our cohort’s tumors, more than half of all cells were stromal cells. While a higher proportion of stromal cells is observed in non-responders compared to responders, this difference is not significant. There may be features in the stromal cell population composition or spatial organization that are predictive of response to therapy and hence should be included in future ML models. Future work exploring stromal cells and incorporating stromal cell features into ML models may be a fruitful direction to expand on this work.

In summary, our study demonstrates how single-cell spatial proteomics, together with ML, can accurately predict advanced melanoma patient response to anti-PD-1 therapy. Both spatial information and immune-rich TME regions proved to be important for ML models to make accurate predictions. This study provides the foundation for expanded analyses and clinical trials that use single-cell spatial proteomics and ML to better predict melanoma patient response to ICB therapy.

## Methods

### Sample information

Tumor samples were collected from Stage IV advanced melanoma patients at Moffitt Cancer Center 1–12 months prior to the start of anti-PD1 immunotherapy. Research involving human participants was conducted in accordance with the Declaration of Helsinki. The study (protocol MCC18583) received approval from Advarra’s external IRB and the Scientific Review Committee at Moffitt Cancer Center. A waiver of consent was granted because the research utilized previously collected tissue samples and retrospective clinical data. All materials were obtained from patients aged 18 years or older. Descriptive information about the patient cohort appears in Table 1. Samples were preserved in formalin-fixed paraffin-embedded (FFPE) tissue blocks and sectioned for multiplex immunofluorescence imaging.

### Multiplex Immunofluorescence

Formalin-fixed and paraffin-embedded (FFPE) tissue samples were immunostained using the AKOYA Biosciences OPAL™ 7-Color Automation IHC kit (Waltham, MA) on the BOND RX autostainer (Leica Biosystems, Vista, CA). The OPAL™ 7-color kit uses tyramide signal amplification (TSA)-conjugated to individual fluorophores to detect various targets within the multiplex assay. Sections were baked at 65 °C for 3 h, then transferred to the BOND RX (Leica Biosystems). All subsequent steps (ex., deparaffinization, antigen retrieval) were performed using an automated OPAL™ IHC procedure (AKOYA). OPAL™ staining of each antigen occurred as follows: heat induced epitope retrieval (HIER) was achieved with Citrate pH 6.0 buffer for 20 min at 95 °C before the slides were blocked with AKOYA blocking buffer for 10 min. The slides were incubated with primary antibodies at room temperature (RT) for 60 min followed by the OPAL™ HRP polymer and one of the OPAL™ fluorophores during the final TSA step. Individual antibody complexes are stripped after each round of antigen detection.

We developed two panels with the following antibodies (summarized in Table S1 and Table S2):

- Lymphoid Panel: iNOS (Thermo Fisher, 4E5, 1:50, dye 570), CD20 (DAKO, L26, HIER-EDTA pH 9.0, 1:150, dye480), PD-L1 (CST, E1L3N, HIER- EDTA pH 9.0, 1:150, dye540), LAG-3 (CST, D2G40, HIER-EDTA pH 9.0, 1:300, dye 690), CD8 (DAKO, C8/ 144B, HIER- EDTA pH 9.0, 1:100, dye520), nNOS (CST, C7D7, HIER-EDTA pH 9.0, 1:300, dye 480), SOX10 (Biocare, BC34, HIER- EDTA pH 9.0, 1:50, dye650) and CD3 (DAKO, Rb poly, HIER- EDTA pH 9.0, dye780).
- Myeloid Panel: iNOS (Thermo Fisher, 4E5, HIER- EDTA pH 9.0, 1:50, dye570), eNOS (CST, D8A6N, HIER- EDTA pH 9.0, 1:50, dye620), N-Cadherin (CST, D4R1H, HIER- EDTA pH 9.0, 1:50, dye480), CD14 (Abcam, LPSR/2386, HIER- EDTA pH 9.0, 1:75, dye540), CD34 (Abcam, EP373Y, HIER- EDTA pH 9.0, 1:250, dye520), MHCII (Dako, M0775, HIER- EDTA pH 9.0, 1:150, dye690), SOX10 (Biocare, BC34, HIER- EDTA pH 9.0, 1:50, dye650) and CD11c (Abcam, EPR4421, HIER- Citrate pH 6.5, 1:150, dye480).

After the final stripping step, DAPI counterstain is applied to the multiplexed slide and is removed from BOND RX for coverslipping with ProLong Diamond Antifade Mountant (Thermo Fisher Scientific). All slides were imaged with the PhenoImager HT Imaging System.

### Quantitative image analysis

Multi-layer TIFF images were exported from InForm (AKOYA) and loaded into HALO (Indica Labs, New Mexico) for quantitative image analysis. The tissue was first segmented into individual cells using the DAPI cell nuclei stain using a proprietary algorithm with the HALO image analysis platform. For each marker, a positivity threshold within the nucleus or cytoplasm was determined per marker based on published staining patterns and intensities for each specific antibody. The per-cell analysis was then exported to provide the marker status (positive or negative) and fluorescent intensity of every individual cell within each image. The resolution of each image was 0.4992 µm per pixel.

### Slide tiling

To capture spatial information at the local tissue level, tiling was performed on all slides to define regions of 1 mm by 1 mm with a minimum of 100 cells per tile. Cells were assigned to each tile based on the location of their centroid, *x*_centroid_ = (*x*_min_ + *x*_max_)/2 and *y*_centroid_ = (*y*_min_ + *y*_max_)/2.

### Cell state proportions

Cell states are defined as the top 20 occurring combinations of protein markers across all cells from all slides, plus individual markers outside of the top 20 combinations. For each cell state, proportions of all cells expressing the respective combination of markers (inclusive of cells expressing those plus other markers) are calculated by dividing cell state counts by the total number of cells within the tile. Cells without any positive markers fall into the “stromal” population (43% of lymphoid panel cells, 52% of myeloid panel cells). Cells can be assigned to multiple cell states, and therefore cell with at least one positive marker is assigned to at least one cell state. Excluded cell states are composed of less than 0.1% of the total cell population in either panel. Tables of all marker combinations and prevalence are included in the project’s GitHub repository in the folders for Fig. 2 and Supplementary Fig. 2 (see Code availability).

### Subpopulation marker proportions

Marker proportions are further analyzed as percentages within defined subpopulations. For each tile, subpopulations are defined as all cells which display a specific marker (ex. all SOX10+ cells, all iNOS+ cells). Within each subpopulation, the proportion of that cell population with every other individual marker is calculated (ex. the proportion of SOX10+ cells that are PD-L1+).

### Recurrent cellular neighborhood (RCN) analysis

Within each tile, cellular neighborhoods are calculated for every cell. Each cell serves as a “seed cell” and its neighborhood is defined as the population of cells within a 60 µm distance. The neighborhood is then quantified as the proportion of each cell state (as defined above in “Cell State Proportions”) within the total cell count of that neighborhood. Using the matrix of all 8 M+ cellular neighborhoods and the 20+ cell state proportion features, we then define recurrent cellular neighborhoods (RCNs) through k-means clustering using the stats package in R. First, we identify the optimal number of clusters (k) via the “elbow method”, increasing k until the within-cluster sum of squares does not decrease. Then, we conduct k-means clustering and assign each neighborhood to an RCN cluster (*k* = 8 RCNs for the lymphoid panel, *k* = 9 RCNs for the myeloid panel). Proportions of each RCN are calculated for each tile to be used as compositional tile features.

### Proximity score features

Proximity scores are calculated to reflect the spatial relationships between cell states across multiple distances. For each pair of cell states within a tile, a distance matrix is calculated containing all the Euclidean distances between each cell of cell state A to every cell of cell state B. To calculate proximity scores, we count the total number of cell state A to cell state B distances less than 20, 30, 60, or 90 µm, then normalize by the sum of the proportions of cell state A and cell state B.

### Ripley’s L features

Ripley’s L features reflect whether cell state pairs show discrete or mixed spatial patterns^54^. Ripley’s K function describes the expected number of cells of cell state A within a specified radius from a typical cell of cell state B. The R package spatstat^55^ is used to calculate Ripley’s K distributions for all cell state pairs within a tile while accounting for edge effects with isotropic border correction. These distributions are then normalized for increased variance at higher radius values by taking the square root of Ripley’s K divided by pi, defined as Ripley’s L. Empirically-derived Ripley’s *L* values (L_iso_) are then compared with theoretical values for randomly-mixed, Poisson-distributed cell state populations (L_theo_) to determine whether the cell state pair is uniformly mixed (L_iso_ ≅ L_theo_), clustered (L_iso_ > L_theo_), or distinct (L_iso_ < L_theo_). Extracted tile features include: (1) Ripley’s *L* values (L_iso_) at 20, 30, 60, and 90 µm, (2) “delta L”, L_iso_ - L_theo_ at 20, 30, 60, and 90 µm representing the degree of spatial clustering (positive) or repulsion (negative) at each distance, and (3) the “cluster score”, integrating delta L from 0 to 100 µm to represent the average clustering/repulsion behavior.

### Univariate statistics

Data analysis and visualization were performed using the R programming language with functions from the stats package and the tidyverse^56^ collection of packages. Wilcoxon rank-sum tests (also known as the Mann–Whitney U test) are performed for each feature, comparing long PFS tiles and short PFS tiles. *P*-values are then corrected for false discovery rate using the Benjamini–Hochberg procedure.

### Machine learning models

Binary classification ML models were created using Light Gradient Boosting Machine^57^ (LightGBM) with PyCaret^58^ in Python to predict tissue sections from long progression-free survival patients (ICB responders) versus tissue sections from short progression-free survival patients (ICB non-responders). We used a leave-one-patient-out cross–validation approach, creating 12 separate models, each with all tiles from one patient left out of the training set and used for testing. This prevents the models from learning patient-specific patterns and artificially improving model accuracy. We aggregated test set prediction metrics across all 12 cross–validation “folds” to report an overall confusion matrix and summary statistics of accuracy, area under the receiver operating characteristic curve (ROC AUC), area under the precision-recall curve (AUPRC), precision, recall, specificity, balanced accuracy, and F1. We conducted feature selection through separate experiments for subsets of compositional and spatial features to identify optimal performance across *n* = 10 iterations. For the lymphoid data, we used 72 features in the Compositional model and 108 features in the Combined and Immune-high models. For the myeloid data, we used 76 features in the Compositional model and 108 features in the Combined model. Final ML experiments (Compositional, Combined, and Immune-high models) were run across *n* = 100 iterations to ensure statistical significance. Each iteration is run with a new random seed, ensuring randomized tree construction.

## Supplementary information

Supplementary figures

Supplementary tables

## Data Availability

The data generated in this study is available for download on Zenodo at 10.5281/zenodo.16898362. Image files are available upon reasonable request.

## Code availability

The code used to generate all computational results and figures is available on GitHub at https://github.com/goeckslab/Melanoma-ICB-mIF.
