# Supplementary material for: Predicting anti-PD-1 immune checkpoint blockade response in melanoma patients with spatially aware machine learning models
Source: NPJ Precis Oncol. 2026 Jan 12;10:56. doi: 10.1038/s41698-025-01250-8 (PMC12877019; doi:10.1038/s41698-025-01250-8)
Supplement: Supplementary file 1 — Supplementary figures [file 41698_2025_1250_MOESM1_ESM.pdf]

## Supplementary Figures

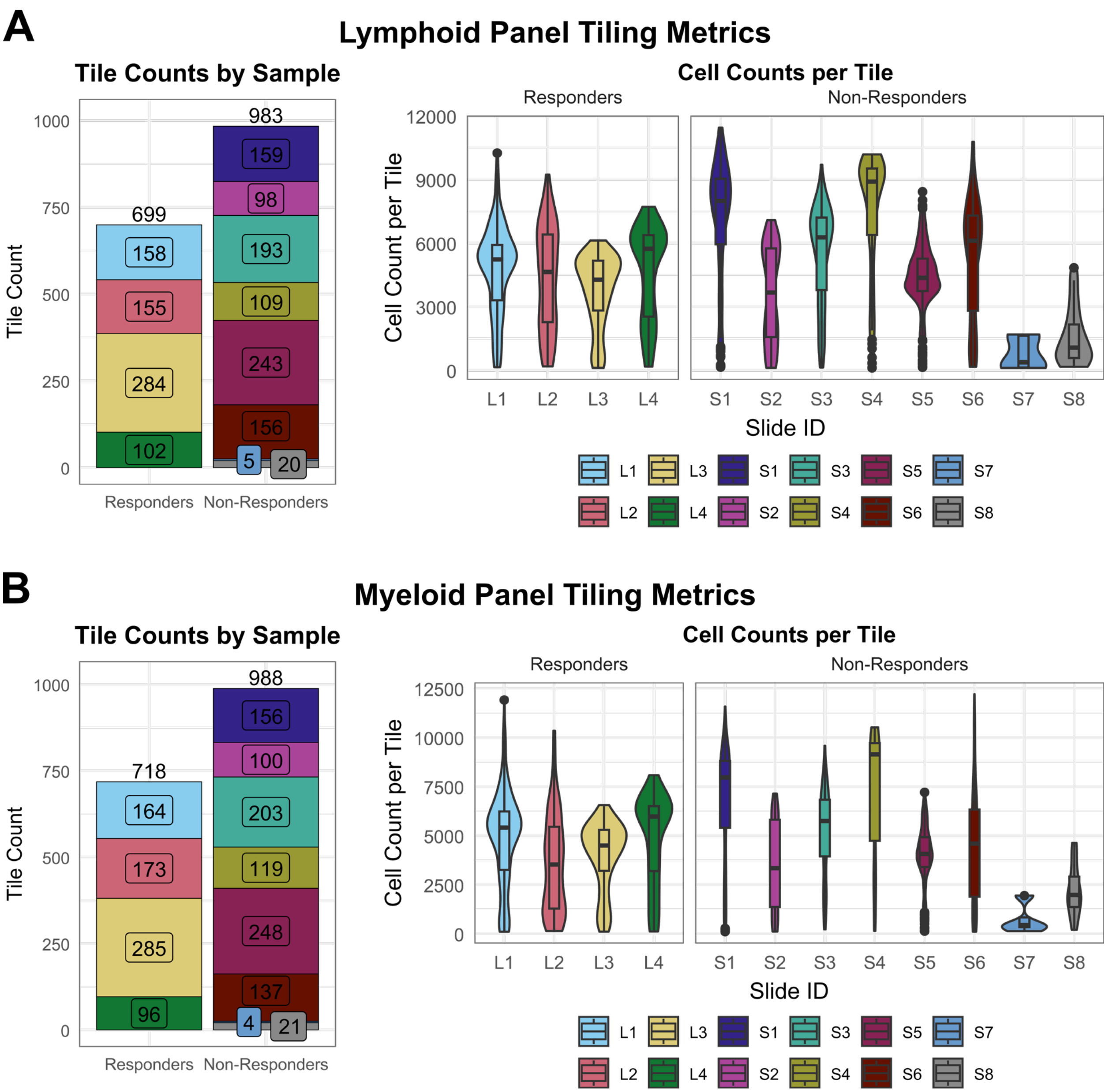


**Supplementary Figure 1. Tile counts by sample and cell counts per tile.** Whole slide images (WSIs) of surgically resected tumors (L1-L4, S1-S6) or tumor biopsies (S7-S8) were separated into 1mm-by-1mm square tiles with a minimum of 100 cells per tile. (**A**) Lymphoid mIF panel WSIs: the number of tiles per resected tumor ranged from 98 to 284, with biopsies contributing 5 (S7) and 20 (S8) tiles (bar chart on the left). In total, the dataset consists of 1,682 tiles: 699 tiles from ICB responder samples and 983 tiles from non-responder samples. Sections of the bar chart are colored by sample corresponding to the legend at the bottom of panel B. Cell counts per tile ranged from a minimum of 100 to over 10,000 (violin/boxplot on the right). Most resected tumor samples have a median of between 4,000 and 6,000 cells per tile, with biopsies showing fewer cells per tile. (**B**) Myeloid panel mIF WSIs: very similar numbers of tiles to lymphoid panel, with 718 from responders and 988 from non-responders (left, 1706 total tiles). Cell counts per tile also showed high concordance with lymphoid panel: 100 to over 10,000 cells per tile (medians around 5,000) and biopsies with fewer cells per tile.

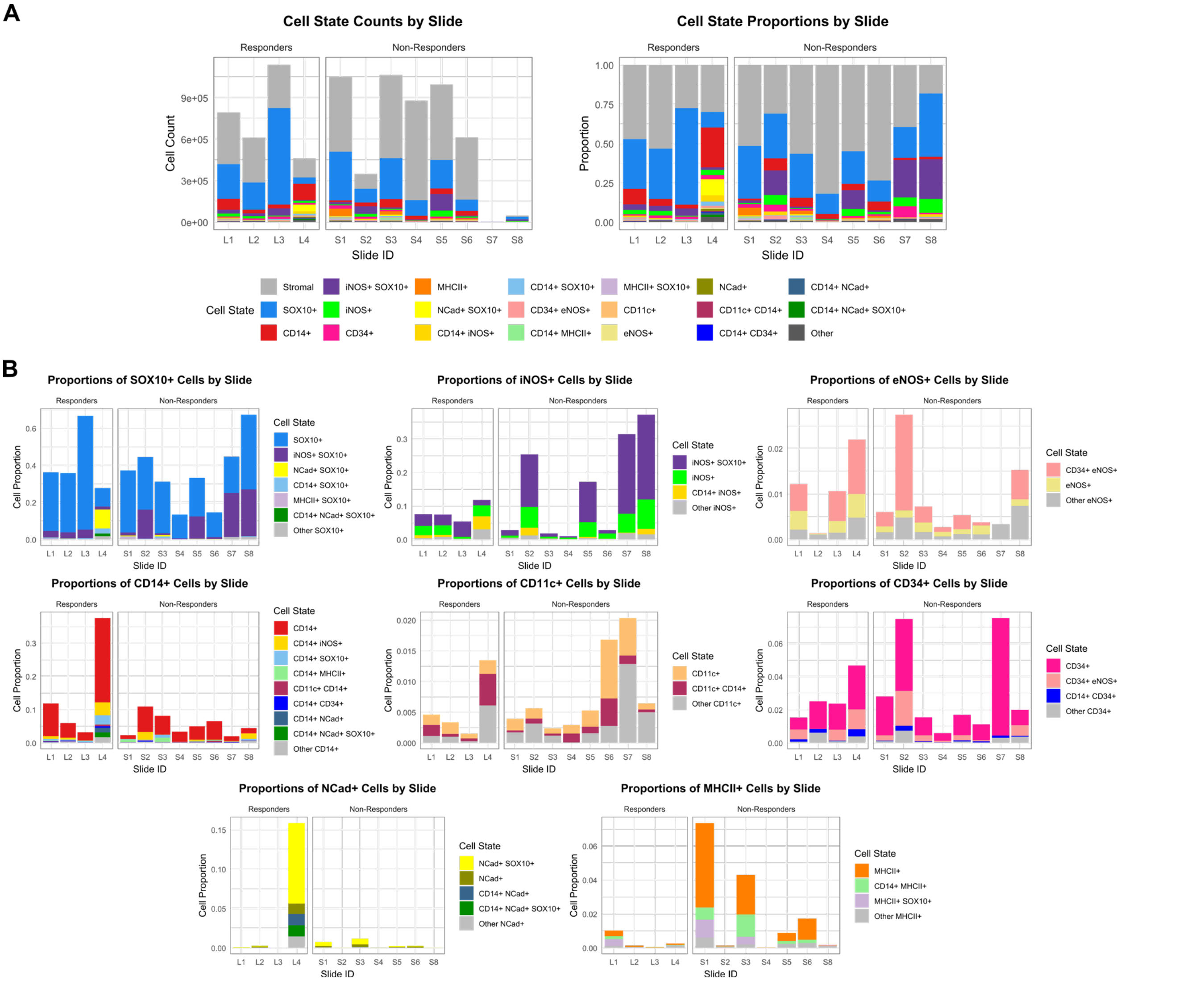


**Supplementary Figure 2. Cell state counts and proportions, myeloid panel.** (**A**) Stacked bar charts displaying cell counts (left) and proportions (right) of each of 20 cell states (plus an "Other" category) by slide. Bars representing ICB responders are displayed on the left (L1-L4) of each chart and bars for non-responders are on the right (S1-S8). Cells lacking any of the eight protein markers are labeled Stromal. (**B**) Stacked bar charts displaying cell state proportions for all cell states containing SOX10, iNOS, eNOS, CD14, CD11c, CD34, NCad, or MHCII (left to right, top to bottom). All cells which contain the specified marker but are not captured within the 20 defined cell states are represented in gray.

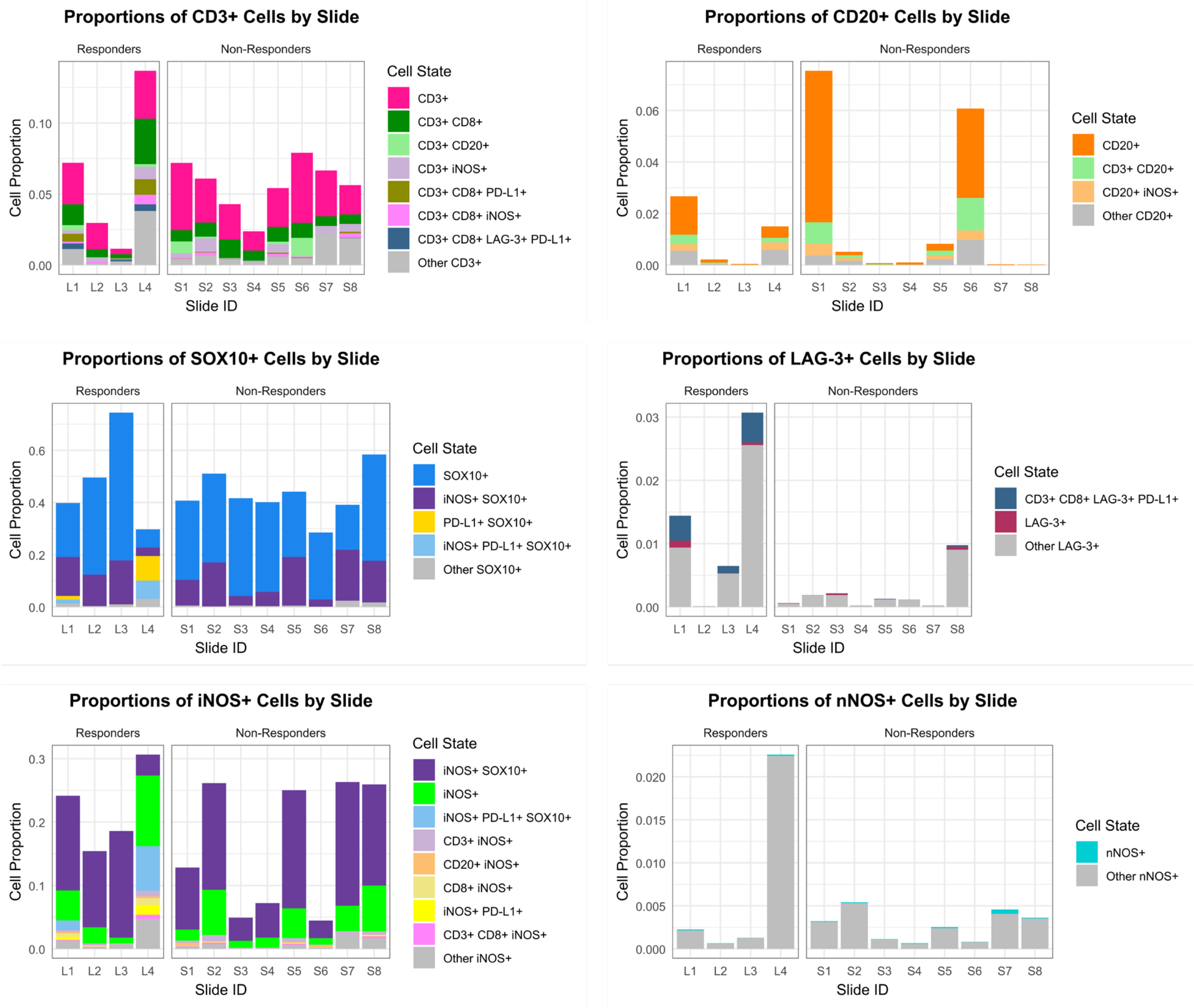


**Supplementary Figure 3. Cell state proportions of select protein markers, lymphoid panel.** Stacked bar charts displaying cell state proportions for all cell states containing CD3, CD20, SOX10, LAG-3, iNOS, or nNOS (left to right, top to bottom). All cells which contain the specified marker but are not captured within the 22 defined cell states are represented in gray.

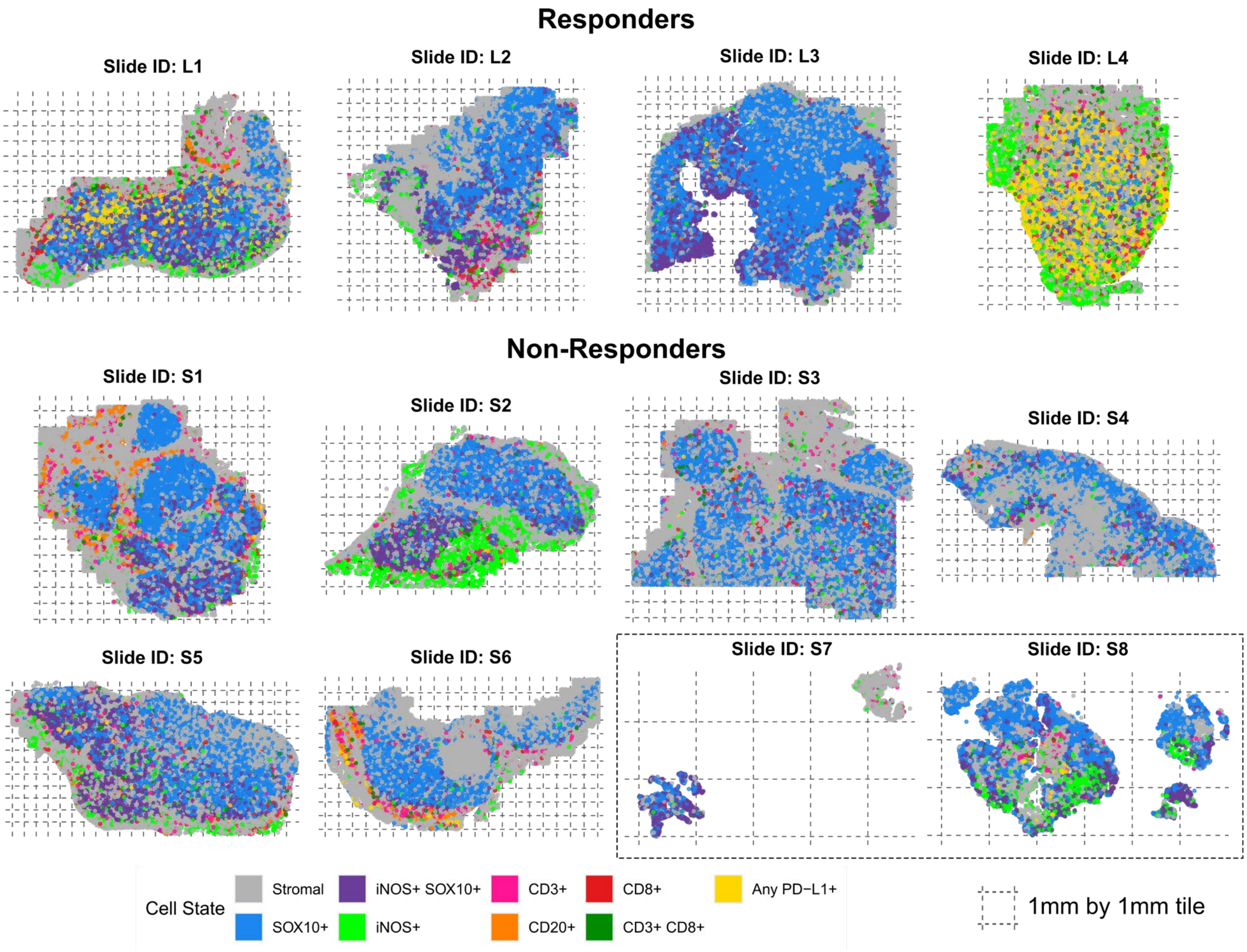


**Supplementary Figure 4. Cell state slide visualizations, lymphoid panel.** Whole-slide visualizations of the spatial distribution of the top 8 cell states (Stromal, SOX10+, iNOS+ SOX10+, iNOS+, CD3+, CD20+, CD8+, CD3+ CD8+) and any PD-L1+ cells (yellow). Cells are plotted by their computed centroids. Dashed lines indicate tile x and y limits. Tiles are 1mm by 1mm in size. The box around samples S7 and S8 indicates these samples are tumor core biopsies; all other samples are tumor resections.

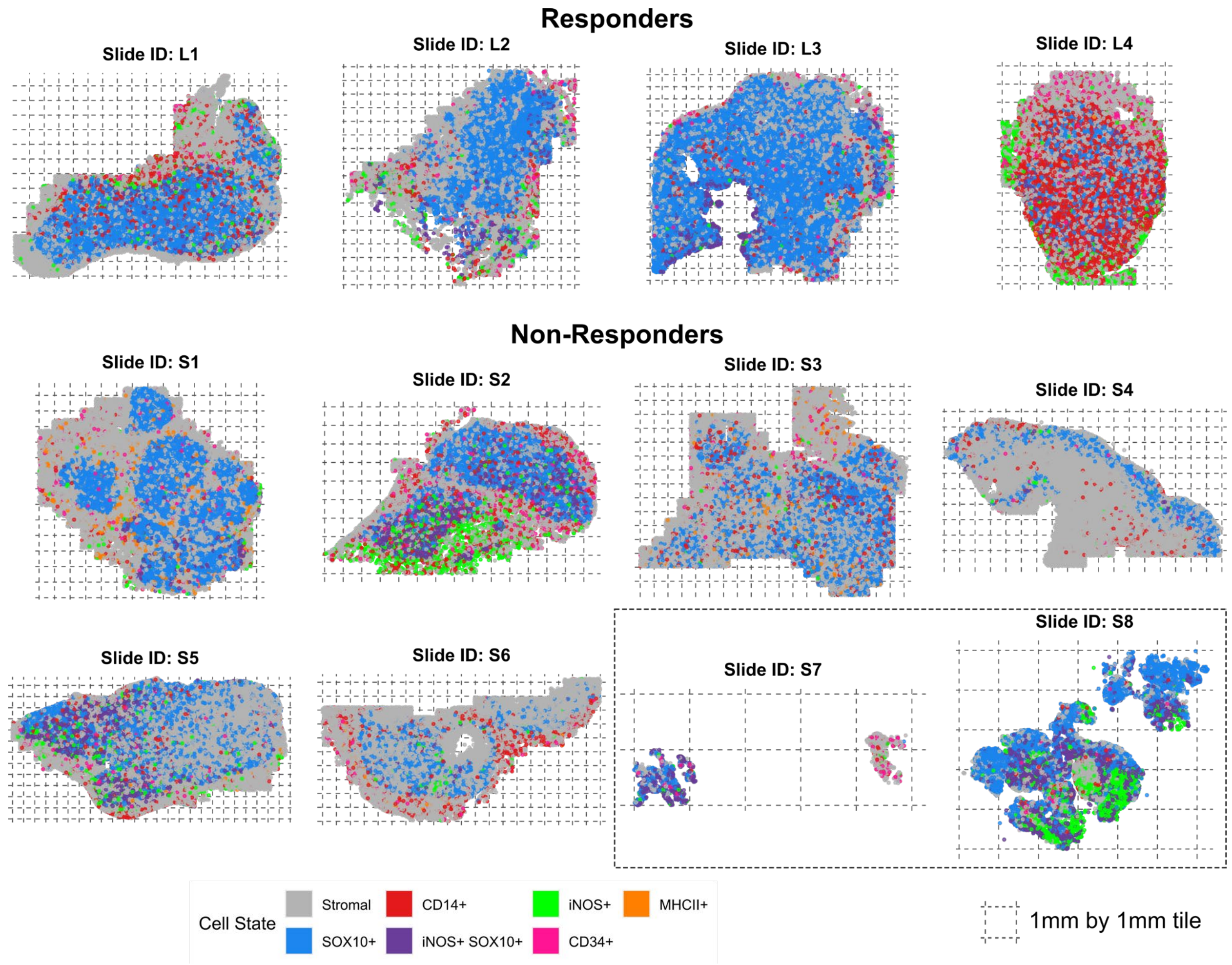


**Supplementary Figure 5. Cell state slide visualizations, myeloid panel.** Whole-slide visualizations of the spatial distribution of the top 7 cell states (Stromal, SOX10+, CD14+, iNOS+ SOX10+, iNOS+, CD34+, and MHCII+). Cells are plotted by their computed centroids. Dashed lines indicate tile x and y limits. Tiles are 1mm by 1mm in size. The box around samples S7 and S8 indicates these samples are tumor core biopsies; all other samples are tumor resections.

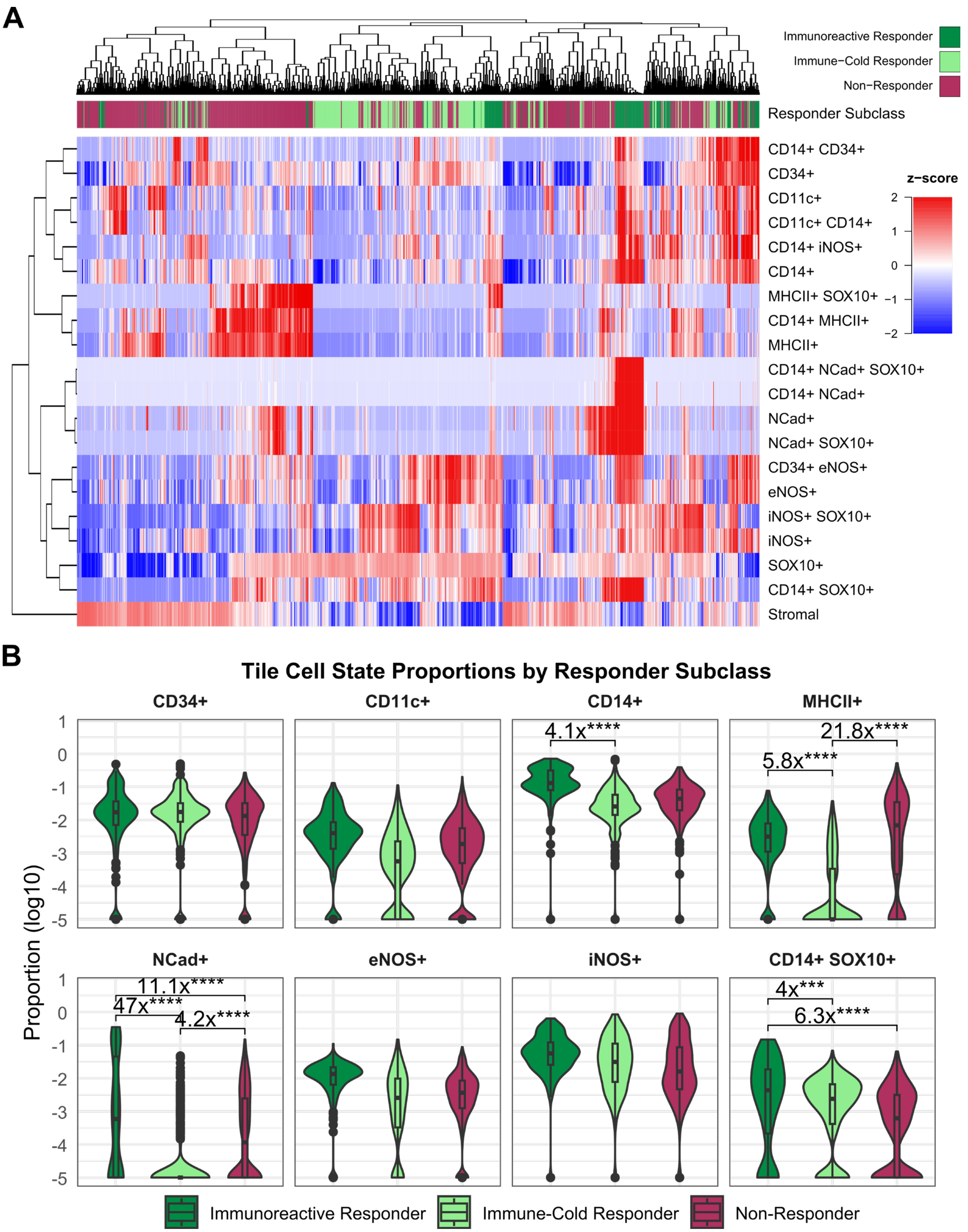


**Supplementary Figure 6. Myeloid panel cell state compositional tile features.** Myeloid panel mIF whole slide images from each patient are separated into 1mm by 1mm tiles and proportions of each of 20 defined cell states are calculated within each tile. (**A**) A heatmap displaying the relative proportions of each cell state (rows) within all 1,706 tiles (columns). Proportions are z-scored across rows to represent the relative expression of each protein (red to blue for high to low expression). The color bar above the heatmap denotes the ICB response subclass of each sample: Immunoreactive Responders (L1/L4), Immune-Cold Responders (L2/L3), and Non-Responders (S1-S8). Rows and columns are clustered by Euclidean distance. (**B**) The tile proportions of CD34+, CD11c+, CD14+, MHCII+, NCad+, eNOS+, iNOS+, and CD14+ SOX10+ cell states (left to right, top to bottom) are displayed according to ICB response subclass: Immunoreactive Responders (L1/L4), Immune-Cold Responders (L2/L3), and Non-Responders (S1-S8). Significant

differences (FDR-corrected Wilcoxon rank-sum p-values) with a fold-change of 4x or greater between response classes are displayed (****p<0.0001, ***p<0.001).

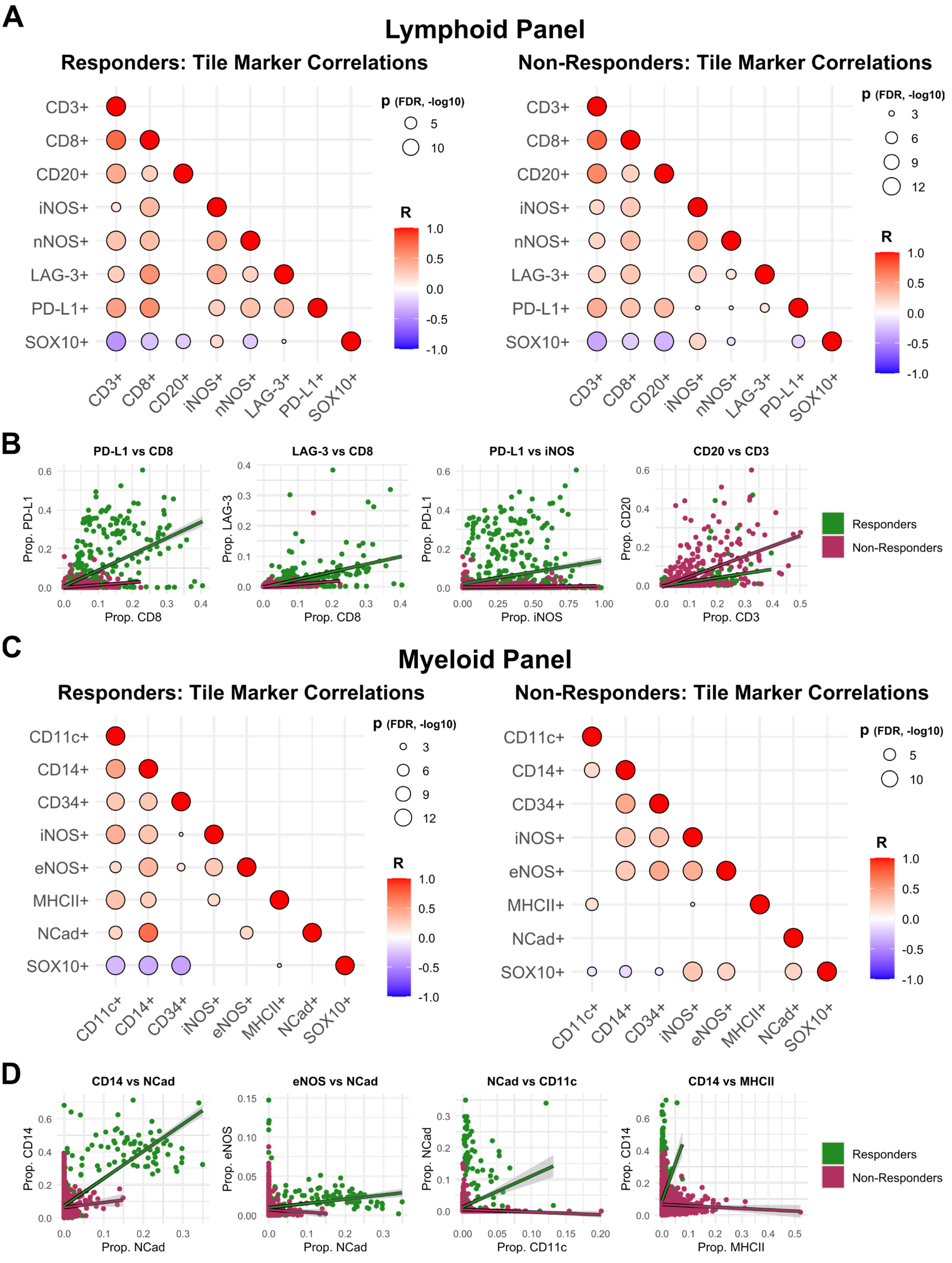


**Supplementary Figure 7. Tile proportion correlations between protein markers differ between responders and non-responders.** Ordinary least squares (OLS) regression was performed between the tile proportions of each individual marker within all responder tiles or all non-responder tiles from WSIs probed with the lymphoid mIF panel (**A-B**) and the myeloid mIF panel (**C-D**). (**A**, **C**)

OLS regression statistics are displayed for each pair of markers, sized by statistical significance (small to large indicating increasing significance by the -log10(p-value)) and colored by Pearson's R correlation coefficient (red to white to blue corresponding to positive correlation, no correlation, and inverse correlation). (**B**, **D**) Scatter plots show differences in correlations for select pairs of individual markers within tiles from ICB responders (green) or non-responders (maroon). Solid lines indicate OLS regression for each response class with shading for 90% confidence intervals. For example, tile proportions of immune checkpoint marker PD-L1 and cytotoxic T cell marker CD8 show a greater degree of correlation within responder tiles versus non-responders (**B**, leftmost plot).

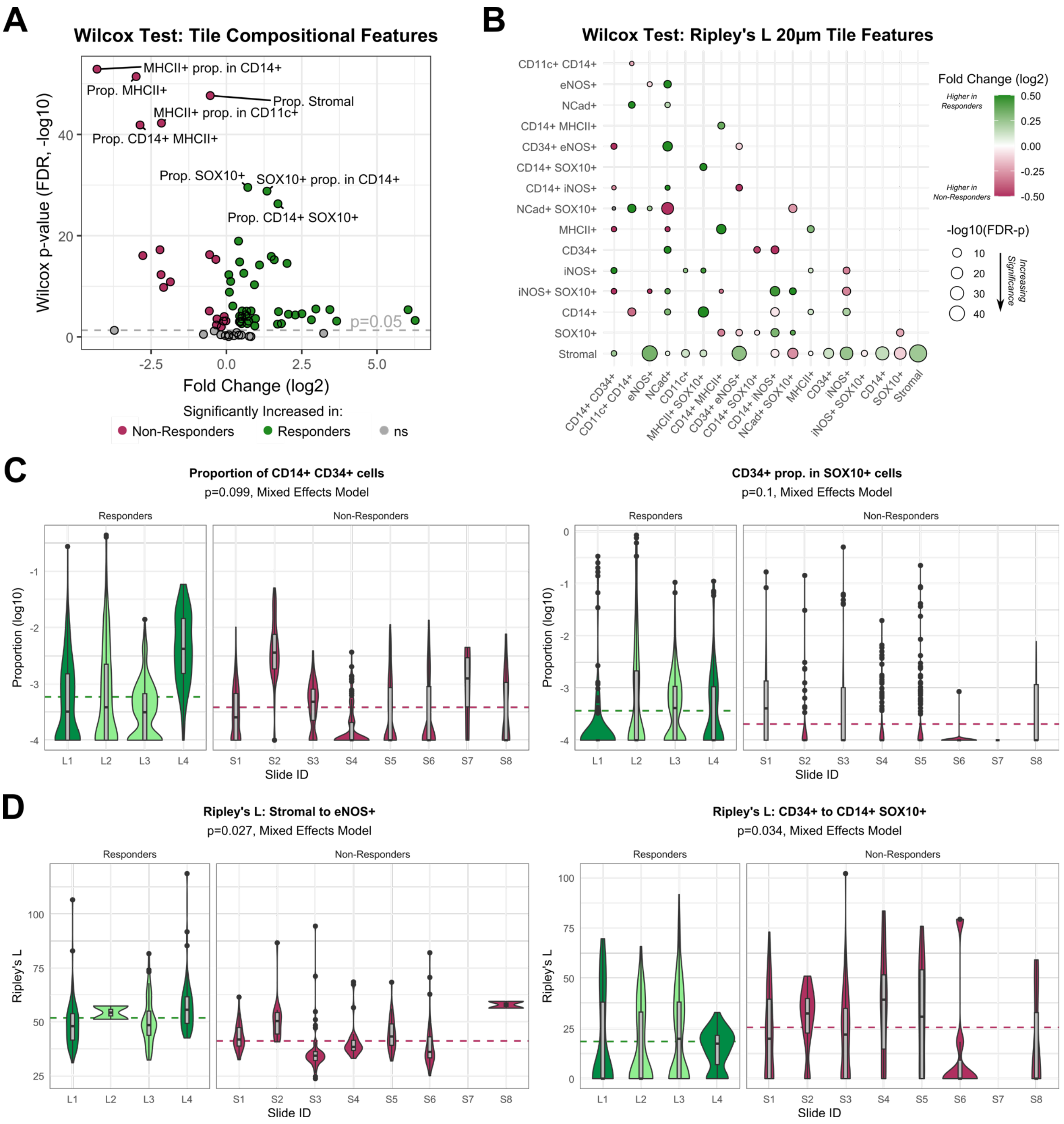


**Supplementary Figure 8. Univariate analysis of ICB responder vs non-responder tile features from myeloid panel.** Wilcoxon rank-sum test (**A**, **B**) and mixed effects modeling (**C**, **D**) were performed to identify compositional and spatial tile features from myeloid panel mIF data in pre-treatment tumor samples capable of distinguishing ICB responders and non-responders. (**A**) Wilcoxon rank sum test was applied to 76 compositional tile features including cell state proportions within the total tile population and proportions of each marker within subpopulations of each other marker. P-values were corrected for false discovery rate (FDR) using the Benjamini-Hochberg procedure. The volcano plot shows increasing significance on the vertical axis (-log10 of the FDR-adjusted p-values) and effect size on the horizontal axis (log2 of the mean fold-change). Positive fold-change values represent features increased within the

ICB responder group. (**B**) Wilcoxon rank-sum test was applied to 169 Ripley's L spatial tile features calculated at 20 μm distances between cells and p-values were FDR-adjusted as above. The bubble plot shows bubbles of increasing size for increasing significance (-log10 of the FDR-adjusted p-values) colored by effect size (log2 of the mean fold-change, with green and maroon indicating increased values in responder or non-responder tiles, respectively) for cell state pairs indicated on the horizontal and vertical axes. Only significant differences are represented with a bubble (if a bubble is missing in the lower diagonal, $p>0.05$). (**C**) To account for slide-to-slide variation within tiles, we also created mixed effects models using slide ID as a random effect to be controlled. None of the 76 compositional features showed significant differences between response groups. The tile distributions of the top two features are displayed by slide and response group on the horizontal axis and log10(proportion) values on the vertical axis. Dotted lines denote the average value of the feature across all tiles of each response class. Mixed effects model p-values displayed in the subtitles are unadjusted. (**D**) Same as (**C**) but for Ripley's L spatial features calculated at 20 μm distances. Out of 91 features, 2 resulted in $p<0.05$ but neither remained significant after FDR-adjustment. Only Ripley's L features with non-missing values in at least 10 of the 12 slides were included in the analysis.

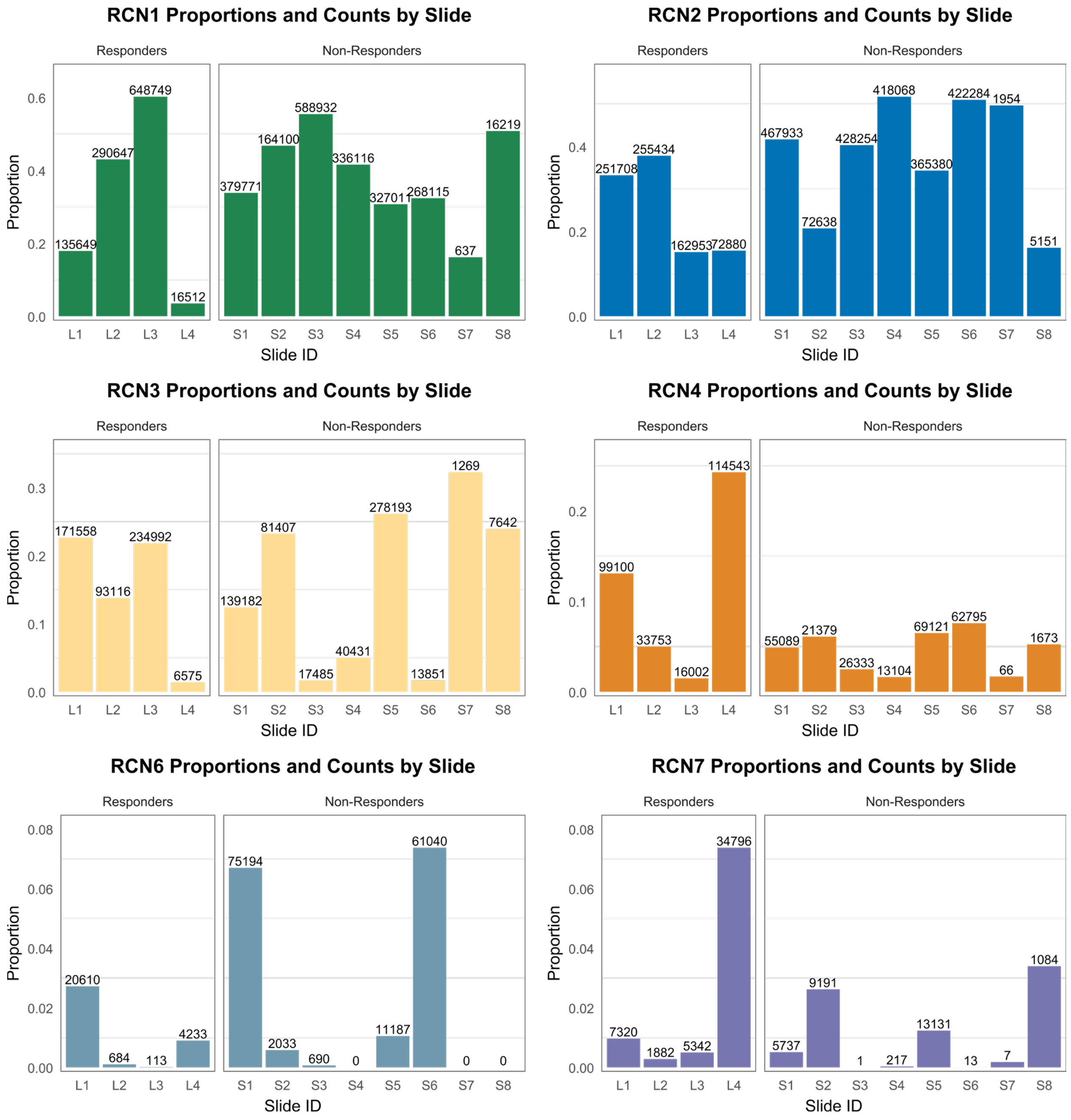


**Supplementary Figure 9. Proportions and counts of recurrent cellular neighborhoods by slide.** Bar charts showing the proportion of RCNs 1, 2, 3, 4, 6, and 7 (left to right, top to bottom) within each slide with labels for total count of RCN neighborhoods displayed above.

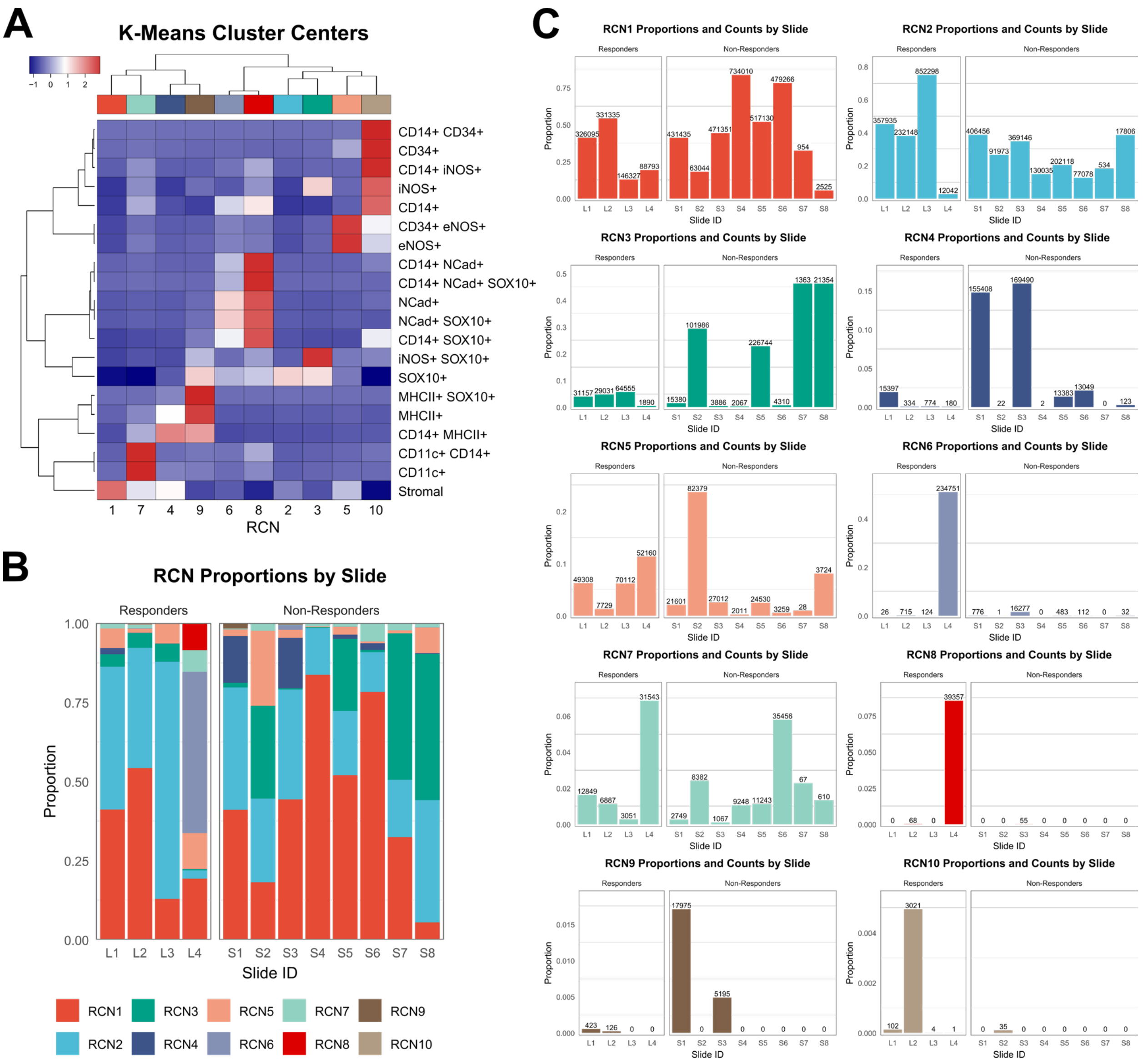


**Supplementary Figure 10. Recurrent cellular neighborhood analysis of myeloid mIF panel.** Recurrent cellular neighborhoods (RCNs) were determined via k-means clustering of all myeloid mIF panel cellular neighborhoods across 12 slides. (**A**) K-means clustering resulted in ten distinct cluster centers which define representative cell state proportion compositions for each RCN. Each column represents an RCN while rows represent cellular neighborhood cell state proportions. Red to blue indicates relatively high to low expression of the corresponding cell state proportion within each RCN. Rows and columns are clustered using Euclidean distance. RCNs are labeled according to size, with RCN1 representing the largest cluster (3,592,265 neighborhoods) down to RCN10, the smallest (3,163 neighborhoods). (**B**) Stacked bar chart showing the proportion of cellular neighborhoods within each slide corresponding to each RCN label. Bars representing ICB responders are displayed on the left (L1-L4) and bars for non-responders are on the right (S1-S8). (**C**) Bar charts showing the proportion of RCN-labeled cellular neighborhoods within each slide with labels for total count of neighborhoods displayed above.

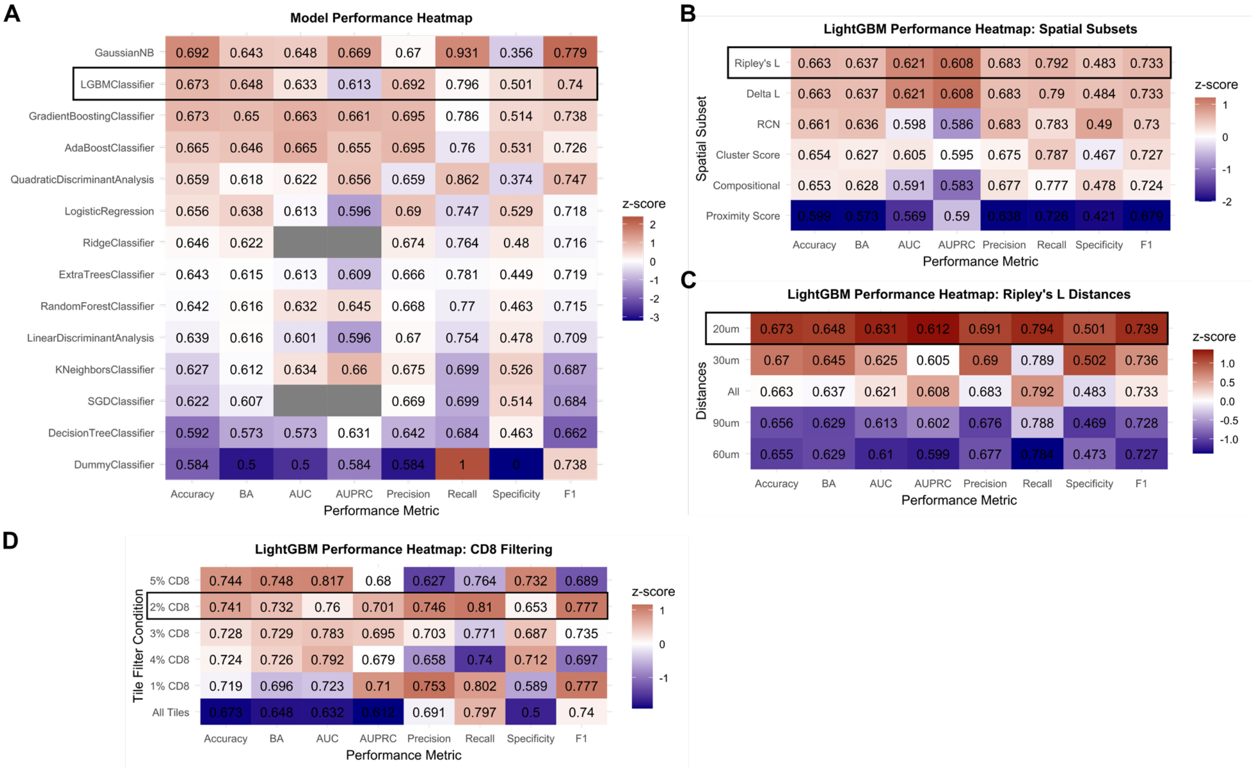


**Supplementary Figure 11. Machine learning model optimization.** To ensure optimal prediction of ICB response by machine learning, we tested multiple ML architectures, feature subsets, and tile filtering conditions. Each model was evaluated based on average performance metrics across 10x iterations of 12-fold leave-one-out cross validation (LOOCV, leaving one of 12 patients out each time) using data from the lymphoid mIF panel. (**A**) Performance results from 13 machine learning models reveals the Gaussian Naïve Bayes (GaussianNB) performs best across many metrics, but specificity is quite low. For this reason, the Light Gradient Boosting Model Classifier (LBGMClassifier) was chosen as it performs similarly to GaussianNB but has much higher specificity. (**B**) To determine which spatial features provided the most useful information for classification performance, we compared performance metrics across models containing all compositional features (proportions and subpopulation proportions) plus either Ripley's L, delta L, RCN proportion, cluster score, or proximity score features (see **Methods** for feature descriptions). Including proximity score features decreased model performance, while model performance was at its best with the inclusion of Ripley's L features. (**C**) To determine the impact of Ripley's L distances on classification performance, we assessed models containing all compositional features plus just Ripley's L features calculated at 20, 30, 60, or 90 µm, or all four distance subsets. Limiting Ripley's L features to values calculated for longer distances (60 and 90 µm) resulted in relatively worse performance versus including just those calculated at 20 and 30 µm, with optimal performance for including just 20 µm Ripley's L features (accounting for close proximity between cell states). (**D**) To determine whether regions with increased immune cell presence are particularly informative for ICB response classification compared to all tissue regions, we created models using our "Combined" feature subset (compositional features, Ripley's L 20 µm features, and RCN proportions) for either all tiles or tiles containing at least 1, 2, 3, 4, or 5% CD8+ cell proportions.  While the strictest filter of 5% CD8+ cells yielded the best overall accuracy, this model suffered from decreases in precision and recall and also resulted in zero tiles from one of our samples, leading us to select the 2% CD8+ filter as the superior model. Every level of CD8 filtering from 1-5% resulted in increased accuracy, balanced accuracy, AUC, AUPRC, and specificity compared to not filtering (All Tiles, 0% filter).

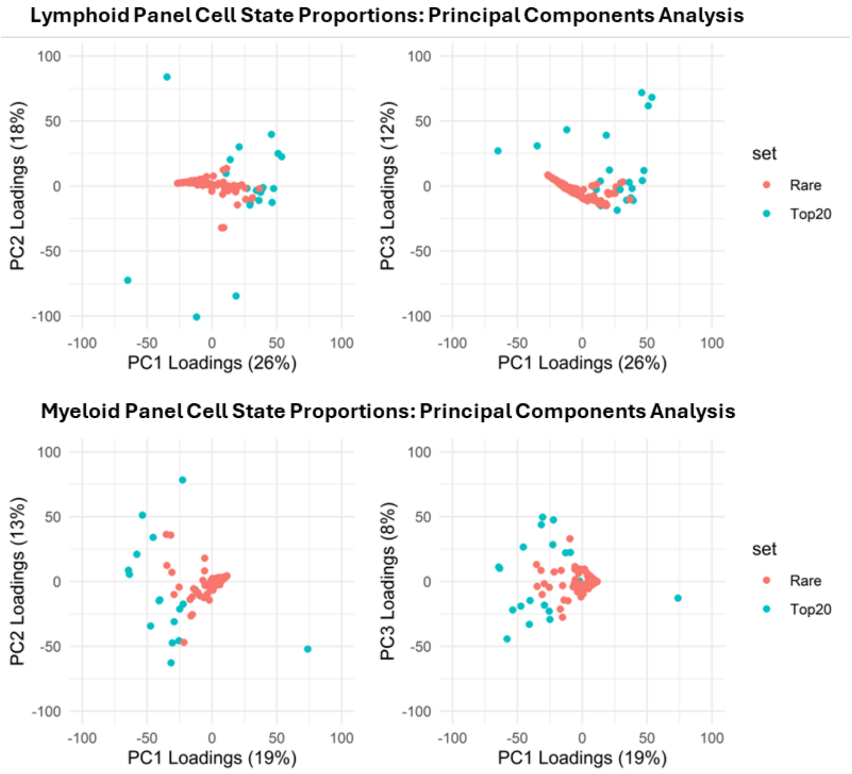


**Supplementary Figure 12. Principal component analysis (PCA) of all cell state proportions.** Principal component loadings of PCs 1, 2, and 3 of cell state proportions within the lymphoid panel (top) and myeloid panel (bottom) show clustering of rare cell states about the origin, indicating low feature contribution to top axes of variance within the data. The top 20 cell states and individual marker cell states are shown in light blue while rarer cell states excluded from our ML models are shown in pink.

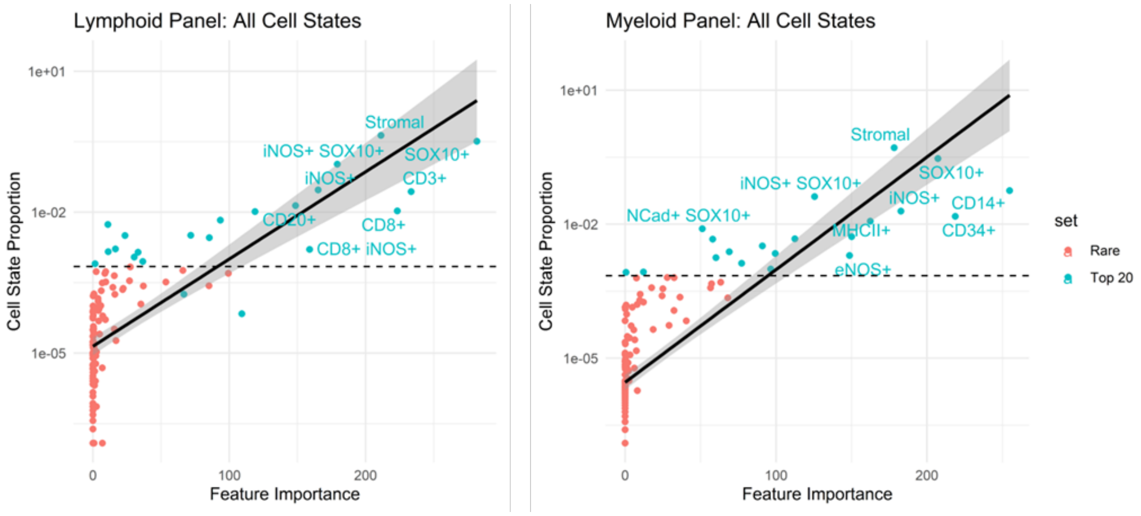


**Supplementary Figure 13. Feature importance of all cell states compositional model.** LightGBM models were trained with all cell state proportions (199 in lymphoid panel, 152 in myeloid panel) and feature split importance was calculated. There is a clear association between cell state proportion and feature importance, with top 20 features and individual marker cell states (light blue) showing increased importance in comparison with rare cell states (pink) excluded in our reported models. The dotted line indicates our proportion threshold for inclusion in the reported models.

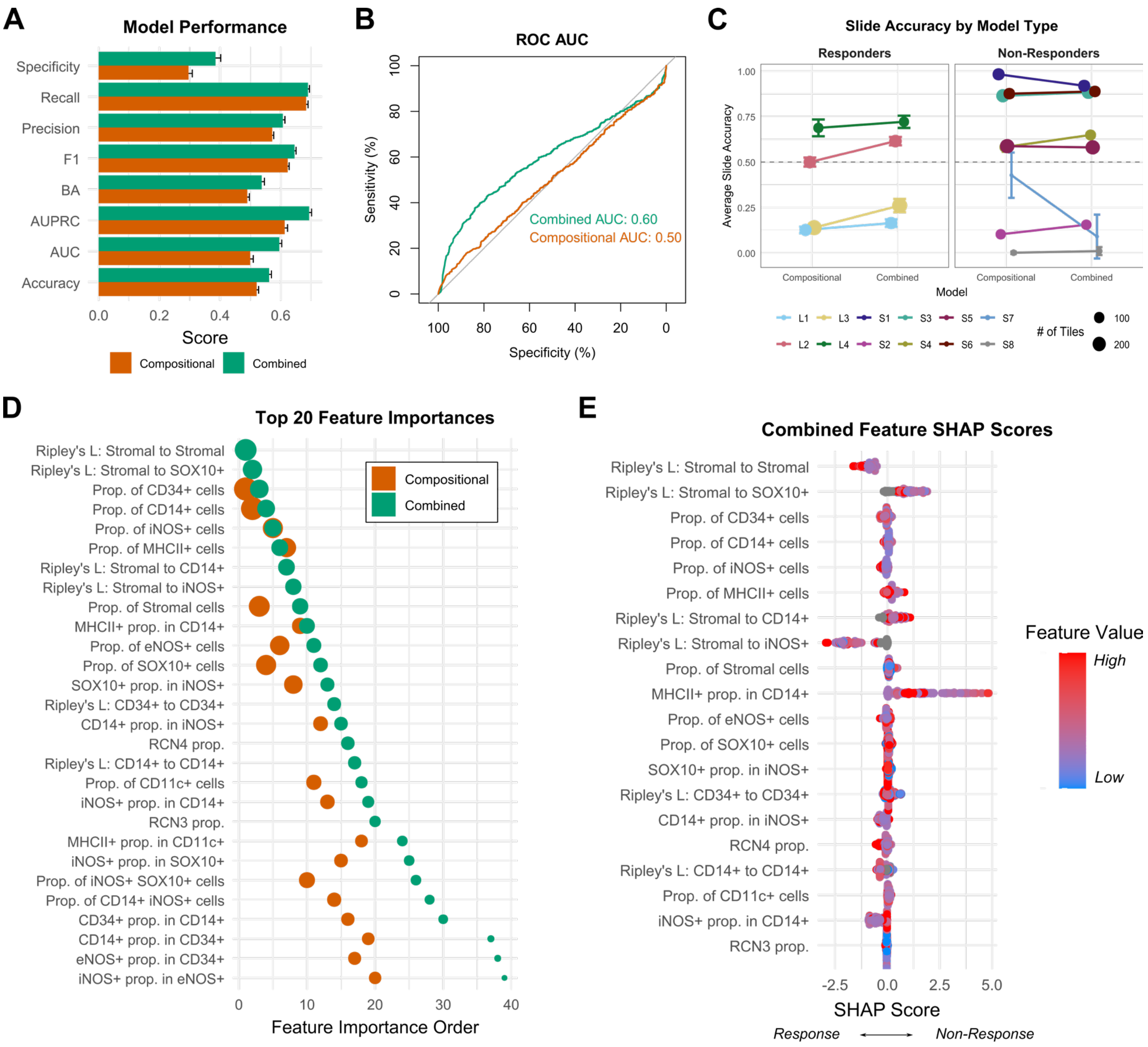


**Supplementary Figure 14. ICB response classification ML models with myeloid mIF panel.** Machine learning model performance metrics calculated from 100 iterations of 12-fold leave-one-out cross validation (LOOCV, leaving one of 12 patients out each time) for the 1) compositional feature only all-tile models ("Compositional") and 2) the combined compositional and spatial feature all-tile models ("Combined") with data from the myeloid mIF panel. (**A**) The Combined model type performed better than the Compositional model. Bars and error bars represent the average and standard deviation of each ML performance metric across 100 iterations of 12x LOOCV. (**B**) The receiver operating characteristic (ROC) curve shows model tradeoffs between sensitivity (true positive rate) and specificity (true negative rate). Area under the curve (AUC) represents the model's ability to distinguish between classes, with higher values corresponding to better performance. The Combined model showed a higher AUC than the Compositional model (AUC=0.60 vs AUC=0.50). (**C**) Tile accuracy by slide for each model type. Points represent average accuracy and error bars represent standard deviation across all 100 iterations of each leave-one-out model. Point size represents the relative number of tiles used for the model test set. The dashed gray line indicates an accuracy of 50%. (**D**) The order of average feature importance values for each model type Data points are colored by model type and sized by their relative average feature importance value. A feature importance order of 1 indicates the feature had the highest feature importance value within that model type. All features within the top 20 average feature importance for either model type are shown. (**E**) Shapley Additive exPlanation (SHAP) scores from a representative Combined model iteration of 12x LOOCV show the relative effect (SHAP score, x-axis) of each feature (y-axis) alongside its relative feature value (red to blue indicates high to low feature values) for each predicted tile (data points). Negative to positive SHAP scores indicate that the associated feature value pushed the model towards predicting ICB response or non-response respectively, while SHAP scores close to zero indicate little to no effect on prediction.
