# Supplementary material for: Predicting anti-PD-1 immune checkpoint blockade response in melanoma patients with spatially aware machine learning models
Source: NPJ Precis Oncol. 2026 Jan 12;10:56. doi: 10.1038/s41698-025-01250-8 (PMC12877019; doi:10.1038/s41698-025-01250-8)
Supplement: Supplementary file 2 — Supplementary tables [file 41698_2025_1250_MOESM2_ESM.docx]

**Table S1: Lymphocyte mIF panel antibody information**

| **Reagent** | **Lot#** | **Product#** | **Opal** | **Clone** | **Dilution** | **Manufacturer** | **AR** |
| --- | --- | --- | --- | --- | --- | --- | --- |
| *Target Antibody 1* ***iNOS*** | YA3808684 | **MA5-17139** | OPAL-570 | 4E5 | **1:50** | Thermo Fisher | **ER2** |
| *Target Antibody 2*  ***CD20*** | 20042864 | **M0755** | OPAL-480 | L26 | **1:150** | Dako | **ER2** |
| *Target Antibody 3* ***PD-L1*** | 18 | **13684s** | OPAL-540 | E1L3N | **1:75** | CST | **ER2** |
| *Target Antibody 4*  ***LAG-3*** | 6 | **15372s** | OPAL-690 | D2G40 | **1:75** | CST | **ER2** |
| *Target Antibody 5* ***CD8*** | 41389238 | **M7103** | OPAL-520 | C8/ 144B | **1:50** | Dako | **ER2** |
| *Target Antibody 6* ***nNOS*** | 2 | **4231s** | OPAL-620 |  | **1:50** | CST | **ER2** |
| *Target Antibody 7* ***SOX10*** | 06022A-2 | **ACI3099A** | OPAL-650 | BC34 | **1:50** | Biocare | **ER2** |
| *Target Antibody 8* ***CD3*** | 41315552 | **A0452** | OPAL-780 | Rb poly | **1:100** | Dako | **ER2** |
|  |  |  | TSA+ Dig |  |  |  |  |

**Table S2: Myeloid mIF panel antibody information**

| **Reagent** | **Lot#** | **Product#** | **Opal** | **Clone** | **Dilution** | **Manufacturer** | **AR** |
| --- | --- | --- | --- | --- | --- | --- | --- |
| *Target Antibody 1* ***iNOS*** | YA3808684 | **MA5-17139** | OPAL-570 | 4E5 | **1:50** | Thermo Fisher | **ER2** |
| *Target Antibody 2* ***eNOS*** | 1 | **35362s** | OPAL-620 | D8A6N | **1:50** | CST | **ER2** |
| *Target Antibody 3*  ***N-Cadherin*** | 6 | **13116s** | OPAL-480 | D4R1H | **1:50** | CST | **ER2** |
| *Target Antibody 4* ***CD14*** | GR3421205-1 | **ab238089** | OPAL-540 | LPSR/2386 | **1:75** | Abcam | **ER2** |
| *Target Antibody 5* ***CD34*** | GR3240236-11 | **ab81289** | OPAL-520 | EP373Y | **1:250** | Abcam | **ER2** |
| *Target Antibody 6*  ***MHCII*** | 20045582 | **M0775** | OPAL-690 | CR3/ 43 | **1:150** | Dako | **ER2** |
| *Target Antibody 7* ***SOX10*** | 06022A-2 | **ACI3099A** | OPAL-650 | BC34 | **1:50** | Biocare | **ER2** |
| *Target Antibody 8*  ***CD11c*** | GR3334379-3 | **ab52632** | OPAL-780 | EP1347Y | **1:100** | Abcam | **ER1** |
|  |  |  | TSA+ Dig |  |  |  |  |
